# Whole-genome sequencing of *Sphingobium baderi* SC-1 and identification of a crucial 3-phenoxybenzoic acid-degrading gene

**DOI:** 10.3389/fmicb.2024.1361335

**Published:** 2024-04-05

**Authors:** Qin Li, Qiao Zhou, Yuan Chen, Kaidi Hu, Montserrat Sarrà, Jianlong Li, Aiping Liu, Likou Zou, Shuliang Liu

**Affiliations:** ^1^College of Food Science, Sichuan Agricultural University, Ya’an, Sichuan, China; ^2^Departament d’Enginyeria Química, Biològica i Ambiental, Escola d’Enginyeria, Universitat Autònoma de Barcelona, Bellaterra, Barcelona, Spain; ^3^College of Resources, Sichuan Agricultural University, Chengdu, Sichuan, China

**Keywords:** *Sphingobium baderi* SC-1, whole-genome sequencing, 3-phenoxybenzoic acid-degrading gene, resting cells, degradation characteristics

## Abstract

As an efficient degradation strain, *Sphingobium baderi* SC-1 can breakdown 3-phenoxybenzoic acid (3-PBA) with high proficiency. To investigate the internal factors that regulate this process, we conducted whole-genome sequencing and successfully identified the pivotal 3-PBA-degrading gene *sca* (1,230 bp). After *sca* was expressed in engineered bacteria, a remarkable degradation efficiency was observed, as 20 mg/L 3-PBA was almost completely decomposed within 24 h. The phenol was formed as one of the degradation products. Notably, in addition to their ability to degrade 3-PBA, the resting cells proficiently degraded 4′-HO-3-PBA and 3′-HO-4-PBA. In conclusion, we successfully identified and validated *sca* as the pivotal enzyme responsible for the efficient degradation of 3-PBA from *Sphingomonas baderi*, providing a crucial theoretical foundation for further explorations on the degradation potential of SC-1.

## 1 Introduction

At present, pyrethroid insecticides (PYRs) account for more than 30% of global insecticides (Quirós-Alcalá et al., 2014) and are widely used in agricultural, forestry and residential pest control (mosquito repellents) (Tang W. et al., 2018); in addition, PYRs have replaced some organophosphorus and carbamate insecticides used in agriculture (Barr et al., 2010). 3-Phenoxybenzoic acid (3-PBA, solubility 24.7 mg/L) is the main intermediate product of PYR metabolism in insects, plants and microorganisms (Sun et al., 2014), and its biotoxicity is greater than that of PYRs. Compared to its parent compounds, 3-PBA is more hydrophilic and mobile. As a consequence, 3-PBA can contaminate food and the environment. As a result of exposure to pyrethroids through the food chain, the parent compound undergoes hydrolysis within the human body, leading to the formation of 3-PBA, which can be detected in human urine (Zhan et al., 2020). Studies have shown that 3-PBA has reproductive and immune toxicity and can cause metabolic disorders in humans (Qi et al., 2012; Sun et al., 2014; Ueyama et al., 2015; Wang et al., 2017). In addition, 3-PBA is recalcitrant to biodegradation and possesses antimicrobial activity, which hinders the mineralization of pyrethroids and increases concern over the elimination of 3-PBA. Therefore, the key to reducing PYR pollution is degrading 3-PBA.

There are many reports on 3-PBA biodegradation, and some 3-PBA-degrading microorganisms have been identified; most of these microorganisms are bacteria, and relatively few are fungi (Topp and Akhtar, 1991; Chen et al., 2012; Jie et al., 2013). Most bacteria can use 3-PBA as the sole carbon source and energy source to degrade 3-PBA through mineralization, while fungi mostly use co-metabolism to degrade 3-PBA (Maloney et al., 1988; Halden and Tepp, 1999; Chen et al., 2011a,b; Jiayuan et al., 2016). In addition, potential degradation pathways of 3-PBA have been proposed by many researchers (Dehmel et al., 1995; Tallur et al., 2008; Wang et al., 2009; Jie et al., 2013; Chen et al., 2015; Birolli et al., 2016a,b; Zhu et al., 2016; Tang J. et al., 2018), who mostly suggested that 3-PBA metabolism begins with the diphenyl ether bond breaking, and oxygen is the key participant (Dehmel et al., 1995; Wang et al., 2014). Oxygenases (monooxygenase and dioxygenase) are important enzymes for the degradation of polycyclic aromatic hydrocarbons. In this process, monooxygenase mainly hydroxylates polycyclic aromatic hydrocarbons, and then dioxygenase mainly cleaves the benzene ring, which is further oxidized into the tricarboxylic acid cycle. Finally, CO_2_ and H_2_O are produced (Liang et al., 2006; Zhao et al., 2017). Therefore, it is important to explore dioxygenases. At present, a limited number of dioxygenase genes and corresponding engineered strains have been reported (Dehmel et al., 1995; Wang et al., 2014); examples include the genes *pobAB*, *pbaA1A2BC*, *d34*, and *dpeAlA2*, which were identified in *Pseudomonas pseudoalcaligenes* POB310, *Sphingobium wenxiniae* JZ-1, *Acinetobacter* sp. 4-D, and *Sphingobium* sp. SC-3, respectively. In our previous work, we isolated a novel strain, *Sphingomonas* sp. SC-1 (specifically identified as *Sphingobium baderi*) from activated sludge, and 300 mg/L 3-PBA could be completely degraded by this strain within 24 h. In addition, the three metabolites of 3-PBA degradation were identified as phenol, catechol and 2-phenoxyphenol. The degradation pathway may involve a variation in the levels of the three metabolites (Jie et al., 2013). However, the intrinsic factors that contribute to this activity remain unclear. In this article, whole-genome sequencing and bioinformatics analysis of strain SC-1 were performed, the key enzyme-encoding genes related to the degradation of 3-PBA were identified, and engineered bacteria were constructed. The studies conducted on PobA and PobB (CAA55400.1 and CAA55401.1) from *P. pseudoalcaligenes* POB310 have provided us with invaluable insights, serving as a crucial reference for the identification of key genes in our research (Dehmel et al., 1995). This work sheds light on the genetic factors involved in the degradation of 3-PBA by *Sphingobium baderi* SC-1, greatly increasing knowledge on the mechanism underlying the action of *Sphingobium baderi* SC-1. Here, the dioxygenase gene, which causes the 3-PBA phenyl ether bond to degrade, was identified in *Sphingobium baderi*.

## 2 Materials and methods

### 2.1 Microorganisms and vectors

*Sphingomonas baderi* SC-1, which was isolated from pesticide plant sludge, was preserved in glycerin at −80°C by the Food Microbiology Laboratory of Sichuan Agricultural University. *Escherichia coli* DH-5α and BL21(DE3) cells, the plasmids pMD18-T and pET28a(+), and Taq polymerase were obtained from Takara (Japan). Restriction endonucleases, T4 DNA ligase and Q5^®^ High-Fidelity DNA polymerase were obtained from NEB, Inc. (USA). The kanamycin (Kan) and isopropyl β-D-1-thiogalactopyranoside (IPTG) used in this study were procured from Beijing Solaibao Technology Co., Ltd. The 3-PBA compound (98% purity) was procured from Tixiai (Shanghai) Chemical Industry Development Co., Ltd.

### 2.2 DNA extraction and whole-genome sequencing

The SC-1 strain was cultured in Luria–Bertani (LB) medium at 30°C until an optical density of 1.0 at OD_600_ was reached. Subsequently, the cell pellets were collected by centrifugation at 4°C (8,000 r/min, 10 min) and washed three times with 0.1 mol/L phosphate-buffered saline (PBS, pH 7). The genomic DNA of strain SC-1 was extracted using the Wizard^®^ Genomic DNA Purification Kit following the manufacturer’s instructions. Prior to library construction, the DNA was quantified using a TBS-380 fluorometer. Sequencing was performed by Shanghai Majorbio Biopharm Technology Co., Ltd. (China), utilizing both PacBio and Illumina MiSeq platforms (Bolger et al., 2014) according to their standard protocols.

(1) For Illumina library construction and sequencing, a minimum of 1 μg of DNA was fragmented to a size of 400 bp using Covaris. Library preparation was carried out utilizing the NEXTflex™ Rapid DNA-Seq Kit, followed by paired-end (2 × 150 bp) sequencing on an Illumina HiSeq X Ten platform.
(2) To construct single-molecule real-time sequencing and sequencing libraries, a minimum of 15 μg of DNA was fragmented into 10 kb fragments using G-tubes. Subsequently, the fragments were purified following PacBio’s instructions, which involved end-filling and ligation to SMRTbell sequencing adapters. The resulting library underwent three rounds of purification with Agencourt AMPure XP beads (0.45×). Then, the fragments were annealed into single-stranded rings and bound to the immobilized polymerase for subsequent real-time detection during the sequencing step.

### 2.3 Bioinformatics analysis and identification of the 3-PBA dioxygenase gene

Bioinformatics analysis was conducted using data generated from the PacBio RS II and Illumina platforms. All analyses were performed on the I-Sanger Cloud platform^1^ provided by Shanghai Maggi Bio. To improve the accuracy of subsequent assemblies and obtain high-quality clean data, reads with lower sequencing quality, higher N proportion, and shorter length were excluded. Canu and HGAP software were used for PacBio data assembly (Koren et al., 2017). The reads were assembled into contigs and further organized into scaffolds to obtain complete chromosome and plasmid genomes. Finally, Illumina sequencing data were utilized to refine the assembly results and determine the circular genome starting site.

Glimmer, GeneMarkS, tRNAscan-SE, and Barrnap were utilized for predicting coding sequences (CDS), plasmid genes, tRNA, and rRNA, respectively (Borodovsky and McIninch, 1993; Besemer and Borodovsky, 2005; Delcher et al., 2007). The predicted CDSs were annotated using sequence alignment tools, such as BLASTP, Diamond, and HMMER, along with the NR, Swiss-Prot, Pfam, GO COG and KEGG databases.

The complete genome sequence of *Sphingomonas baderi* SC-1 was functionally annotated to identify dioxygenase genes. Then, a BLAST search in NCBI was performed to identify the 3-PBA degradation enzyme-encoding gene based on sequence comparison. Phylogenetic trees of the target genes were constructed using MEGA 5.0 and analyzed with 1,000 replicates per clade.

### 2.4 Cloning and validation of dioxygenase genes

*Sphingomonas baderi* SC-1 cells were cultured in LB media. Target genes without the native secretion signal were amplified by PCR from genomic DNA using corresponding primers (Supplementary Table 1). The resulting DNA fragments were subcloned and inserted into the pMD18-T plasmid, transformed into *E. coli* DH-5α cells, and sequenced for verification. Subsequently, the PCR products were cloned and inserted into the pET28a(+) plasmid at the *Bam*HI and *Hin*dIII restriction sites and expressed in *E. coli* BL21 (DE3) to produce dioxygenases. The 1% (v/v) genetically engineered bacteria were grown overnight in 100 ml of LB media supplemented with 40 μg/ml Kan at 37°C, and the resulting culture was used for subsequent experiments on dioxygenase expression.

After 1.0% (v/v) of the culture was inoculated into 100 ml of LB medium supplemented with 40 μg/ml Kan, the mixture was incubated at 37°C and shaken at 160 rpm for 3.0 h until an optical density (OD_600_) of 0.5∼0.6 was reached. Subsequently, induction of expression was achieved by adding IPTG to a final concentration of 1 mmol/L and maintaining the temperature at 20°C for 16 h. Following this, 3-PBA stock solution was added, to achieve a final concentration at 20 mg/L, and the mixture was incubated with shaking at 37°C and rotation at approximately 160 rpm for an additional 48 h. The samples (1 ml) were collected at designated time points and the 3-PBA residue was detected (the reaction was terminated using acetonitrile) (Li et al., 2016). Subsequently, the samples were mixed with 4 ml of acetonitrile and subjected to ultrasonic extraction (40 kHz) for 30 min, followed by centrifugation at 12,000 r/min for 10 min. After supernatant was passed through a filter membrane (0.22 μm) in the organic phase, the filtrate was utilized to assess the percentage degradation of 3-PBA using high-performance liquid chromatography (HPLC) equipped with a Gemini C18 column (pore size, 100 Å; particle size, 5.0 μm; dimensions, 150 mm × 4.60 mm), an injection volume of 10 μl, a column temperature of 25°C, and a mobile phase consisting of an acetonitrile-ultrapure water mixture (55:45 v/v). The flow velocity was maintained at 0.7 ml/min, and the UV detection wavelength was set at 210 nm. The *E. coli* BL21(DE3)-pET-28a(+) strain was used as a control. Moreover, the effects of inoculation amount (0.5%, 1.0%, 1.5%, 2.0%, and 2.5%), preinduction culture time (1.0, 1.5, 2.0, 2.5, and 3.0 h) and induction temperature (16, 18, 20, 22, and 24°C) on the percent degradation of 3-PBA were explored using the percent degradation of 3-PBA as an indicator (see Supplementary Figure 1 for detailed results). Then, the Box-Behnken Center combined experimental design was used to investigate the factors that affect the percent degradation of 3-PBA based on a single factor. The degradation time was set at 6 h.

According to the optimization described above, the inoculation amount was 1.5%, the preinduction culture time was 2.50 h, and the induction temperature was 20°C (see Supplementary Tables 2, 3 and Supplementary Figure 2 for detailed results). These results were used in subsequent experiments on dioxygenase expression. The degradation of 3-PBA in resting cells, whole-cell lysates, cell lysate supernatants, and cell debris suspensions was subsequently investigated.

For the resting cells, after IPTG (1 mmol/L) was used for the induction of expression at 20°C for 16 h, 100 ml of culture was centrifuged (8,000 r/min, 10 min) at 4°C, as described above. The obtained biomass was washed once using 0.1 mol/L PBS, 3-PBA stock solution was added to the resuspended cells (resting cells, 3.3 × 10^10^ CFU/ml) with 10 ml of 0.1 mol/L PBS, to achieve a final concentration at 20 mg/L, and the mixture was incubated with shaking at 37°C and 160 rpm.

For the whole cell lysate, after IPTG (1 mmol/L) was used to induce expression at 20°C for 16 h, 100 ml of culture was centrifuged (8,000 r/min, 10 min) at 4°C, as described above. The obtained biomass was washed once using 0.1 mol/L PBS, and the cell resuspended in 10 ml of 0.1 mol/L PBS was ready for ultrasonic wall disruption (65 W, working for 2 s at intervals of 2 s, 25 min) in an ice bath. Then, 900 μl of whole-cell lysate and 100 μl of 3-PBA stock solution were mixed, to achieve a final concentration at 20 mg/L, and the mixture was incubated with shaking (160 rpm) at 37°C and 160 rpm.

For the cell lysate supernatant and cell debris suspension, the whole cell lysate as described above was centrifuged (8,000 r/min, 10 min) at 4°C, and the supernatant was collected as a cell lysate supernatant. The precipitate was washed once using 0.1 mol/L PBS, and the precipitate was resuspended in 10 ml of 0.1 mol/L PBS as a cell debris suspension. Then, 900 μl of cell lysate supernatant/cell debris suspension and 100 μl of 3-PBA stock solution were mixed, to achieve a final concentration at 20 mg/L, and shaking (160 rpm) was continued at 37°C and 160 rpm.

### 2.5 Characterizing 3-PBA degradation in resting cells

#### 2.5.1 Time-course degradation of 3-PBA in resting cells

As described in section “2.4 Cloning and validation of dioxygenase genes,” 3-PBA was spiked into the resuspended resting cell with 10 ml of 0.1 mol/L PBS up to a final concentration of 20 mg/L, and shaking (160 rpm) was continued at 37°C and 160 rpm. Then, the samples were taken at designated times (0, 0.5, 1, 1.5, 2, 2.5, 3, and 4 h) and the 3-PBA percent degradation was assessed using HPLC.

#### 2.5.2 Metabolite identification

The products of 3-PBA degradation by *sca*-engineered resting bacterial cells were analyzed using HPLC and gas chromatography-mass spectrometry (GC-MS).

A standard phenol solution with a concentration of 10 mg/L was prepared and introduced into the 2-h extract of 3-PBA degraded by resting cells. HPLC was then employed to detect the normalized samples according to the corresponding criteria. The HPLC detection conditions for phenol and standardized samples were identical to those described in section “2.4 Cloning and validation of dioxygenase genes.”

Then, acetonitrile extracted from the degradation of 3-PBA by resting cells was collected (5 ml solution) at various time intervals. The sample was concentrated under a stream of nitrogen, followed by redissolution in 500 μl of ethanol. After centrifugation at 12,000 r/min for 10 min, the supernatant was filtered through an organic phase filter membrane (0.22 μm) for GC-MS analyses.

The following improved GC-MS detection parameters were employed: chromatography was conducted using an HP-5MS capillary column (30 m × 0.25 mm × 0.25 μm) coated with a 5%-phenyl-methylpolysiloxane stationary phase, and helium was used as the carrier gas. The inlet temperature was set at 260°C, with a sample size of 1 μl and a flow rate of 1.0 ml/min. A programmed temperature method was employed, starting at 80°C for 1 min, followed by a temperature increase from 10°C/min to 260°C in 15 min without any shunt injection. Full scan analysis was performed.

#### 2.5.3 The optimum temperature and pH

To investigate the effect of temperature on the degradation of 3-PBA by *sca*-engineered bacterial resting cells, 20, 25, 30, 35, 40, 45, 50, and 55°C were applied. Reaction system (1 ml): 900 μl of 0.1 mol/L PBS was used to resuspend the resting cells, and the volume of 3-PBA stock solution was 100 μl, to achieve a final concentration at 20 mg/L. After the oscillatory reaction was performed for 2 h in a 160 r/min water bath at the temperature gradient described above, acetonitrile was added to quench the reaction.

The resting cells were placed in 0.1 mol/L citrate buffer at pH 3.5–6.0, phosphate buffer at pH 6.0–8.0, Tris–HCl buffer at pH 8.0–10.0, and NaHCO_3_-NaOH buffer at pH 10.0–13.0. The effect of varying pH on the degradation of 3-PBA in resting cells was investigated. For the reaction system (1 ml), 900 μl of buffer at different pH values was used to resuspend the resting cells, and 100 μl of 3-PBA stock solution was added, to achieve a final concentration at 20 mg/L. After the reactants were placed in a water bath at 160 r/min for 2 h, acetonitrile was added to stop the reaction.

#### 2.5.4 Temperature and pH stability

The temperature stability of 3-PBA degradation by resting cells was investigated. The resting cells were placed at 20, 30, 35, 40, and 50°C for 1 h and then subjected to a 35°C and 160 r/min water bath for an oscillating reaction for 2 h. The reaction was terminated using acetonitrile.

The pH stability of 3-PBA degradation by resting cells was investigated with different pH buffers (citrate buffer, 4.0–5.0; phosphate buffer, 6.0–8.0; Tris–HCl buffer, 8.5–10.0; and NaHCO_3_-NaOH, 11.0). The suspended resting cells were placed at 35°C for 1 h, after which 3-PBA stock solution was added to a water bath, to achieve a final concentration at 20 mg/L, at 35°C and 160 r/min for the oscillatory reaction. The samples were collected every 2 h, and acetonitrile was added to terminated he reaction.

#### 2.5.5 The effect of metal ions

The effect of different concentrations of metal ions on the degradation of 3-PBA in resting cells was investigated. The mother liquors of ZnSO_4_⋅7H_2_O, MnSO_4_⋅7H_2_O, MgSO_4_⋅H_2_O, CuSO_4_⋅5H_2_O, FeCl_3_, FeSO_4_⋅7H_2_O, NaCl, KCl, BaCl_2_, CaCl_2_⋅5H_2_O, CdCl_2_⋅2.5H_2_O, and Pb(CH_3_COO)_2_⋅3H_2_O were added to the reaction system, and the final concentrations of the metal ions were 1 and 5 mmol/L. After the reactants were oscillated in a water bath at 35°C and 160 r/min for 1 h, acetonitrile was added to efficiently quench the reaction.

#### 2.5.6 The effect of chemical reagents

Chemical solvents have the potential to perturb the conformation of enzymes, thereby impeding their catalytic activity. The anionic surfactant SDS, for instance, can disrupt the non-covalent interactions among enzyme molecules, thereby exerting an influence on enzyme activity. The higher polarity of organic solvents may impede the ingress of water molecules into the active center of the enzyme, thereby attenuating enzymatic activity (Sadana, 1988). The effect of different concentrations of chemical reagents on the degradation of 3-PBA in resting cells was thus investigated. Methanol, ethyl alcohol, isopropyl alcohol, polyethylene glycol 400, acetone, n-hexane, and dimethyl sulfoxide were added to the reaction system, resulting in final concentrations of 1% and 5%, respectively. Urea, EDTA, SDS, DTT, and CTAB were added with final concentrations of 1 and 5 mmol/L, respectively. After the reactants were oscillated in a water bath at 35°C and 160 r/min for 1 h, acetonitrile was added to was effectively quench the reaction.

#### 2.5.7 Determination of substrate tolerance

To investigate the tolerance of resting cells to the substrate 3-PBA, 3-PBA was added to the reaction system at final concentrations of 10, 20, 30, 40, 50, 60, 70, and 80 mg/L. After the reactants were subjected to oscillation in a water bath at 35°C and 160 r/min for 2 h, acetonitrile was added to terminate the process.

#### 2.5.8 The effect of cell concentration

To investigate the effect of the resting cell concentration on the percent degradation of 3-PBA, 3-PBA stock solution was added to the different concentrations of resting cells, to achieve a final concentration at 20 mg/L. After the reactants were subjected to oscillation in a water bath at 35°C and 160 r/min for 2 h, acetonitrile was added to quench the reaction.

#### 2.5.9 Substrate specificity determination

The specificity of *sca*-engineered bacterial resting cells for the degradation of substrates was investigated. 4′-HO-3-PBA, 3′-HO-4-PBA, phenol, catechol, gallic acid, protocatechuic acid, and flusulfamide ether were added to the conversion reaction system at a final concentration of 20 mg/L. The reaction was quenched by the addition of acetonitrile after 2 h in a water bath at 35°C and 160 r/min. The HPLC detection conditions for flusulfamide ether were as follows: the mobile phase consisted of a mixture of acetonitrile and phosphoric acid water at pH 2.5 (65:35, v/v), the flow rate was set to 1 ml/min, the column temperature was maintained at 30°C, the detection wavelength was set to 290 nm, and an injection volume of 20 μl was used.

### 2.6 Data analysis

The percent degradation was calculated according to the following equation:


$$\mathrm{percent}\mathrm{degradation}\left(\%\right)=\left(1-C/C_{0}\right)\times 100$$

where *C* represents the residual concentration of substrates in sample solutions (mg/L) and *C*_0_ is the initial concentration of substrate (mg/L).

The mean and standard deviation of the percent degradation in each trial were calculated and subjected to ANOVA. Statistical significance was determined using SPSS V22.0.

## 3 Results

### 3.1 Bioinformatics analysis of whole-genome sequences

#### 3.1.1 Quality control analysis of DNA and sequencing data

The main DNA band was >10 kb, with no visible trailing, meeting the requirements for constructing sequencing databases (Supplementary Figure 3). The quality control results of the Illumina sequencing data are shown in Supplementary Table 4 and Supplementary Figure 4. The Q20 and Q30 values were 97.48% and 93.12%, respectively, and after quality control, they reached 98.22% and 94.15%, respectively. There were 85,064 reads and a base length of 1,144,377,555 bp, with the longest read length of 161,883 bp and an average read length of 13,453.14 bp based on the quality control data of the PacBio reads (Supplementary Figure 5).

#### 3.1.2 Genome assembly and evaluation results

The SC-1 genome has 1 chromosome and 6 plasmids, with a total size of 4,374,142 bp and an average GC content of 63.53%. The chromosome size was 3,601,664 bp, and the GC content was 63.79%. The sizes of plasmids A∼F were 349,403, 16,766, 97,252, 77,650, 65,982, and 12,840 bp, respectively, and the GC contents were 62.01%, 63.76%, 61.81%, 62.28%, 61.49%, and 60.90%, respectively (Supplementary Table 5). The GC content is an important index of the genome of each species and can be used to judge whether a sample contains other genome contaminations. As shown in Supplementary Figure 6, most of the GC-depth spots in the SC-1 genome were concentrated within a relatively narrow range, indicating that the sample DNA was not contaminated. K-mer analysis was used to select high-quality sequencing regions in the Illumina sequencing reads. The 17-mer was used as a parameter and followed a Poisson distribution with a single main peak, indicating that the sample DNA was not contaminated by other sources of DNA (Supplementary Figure 7). The genomic information has been submitted to the National Center for Biotechnology Information (NCBI) database (Accession ID: SAMN35671193).

#### 3.1.3 Genome analysis and genome function annotation

As shown in Supplementary Table 6, 4,432 genetic sequences that represented 88.93% of the genome were predicted for functional annotation analysis, and 1,488 genes > 1,000 bp were identified (Supplementary Figure 8). Regarding the RNA genes, there were 51 tRNAs (Supplementary Table 7) and 6 rRNAs (2 16S rRNAs, 2 23S rRNAs, and 2 5S rRNAs). The distributions of protein-coding sequences (CDSs), RNA genes, COG functions, and GC content in the genome are shown in a genome circle map (Supplementary Figure 9). Genome function annotation was demonstrated mainly through comparison with six biological databases, including Non-redundant protein database (NR), Swiss-Prot database, Pfam database, Gene Ontology database (GO), Clusters of Orthologous Groups of proteins database (COG), and Kyoto Encyclopedia of Genes and Genomes database (KEGG), as shown in Supplementary Figure 10 and Supplementary Table 8.

### 3.2 Screening and validation of dioxygenase genes

*Sphingomonas* sp. can metabolize aromatic compounds and synthesize valuable extracellular polymers, which is highly valuable for research in many fields, such as environmental science and food and industrial production (Lee et al., 2001; Yabuuchi et al., 2011). Eighteen dioxygenase genes were screened based on the gene annotation results (Supplementary Table 9); from these genes, two genes were obtained and designated *sca* (phenoxybenzoate dioxygenase subunit alpha, pC_gene0033) and *scb* (phenoxybenzoate dioxygenase subunit beta, pC_gene0035). Both genes were situated on plasmid C, and *sca* and *scb* were 1,230 and 960 bp in length, respectively. The encoded proteins Sca and Scb were compared with the α subunit (PobA, CAA55400.1) and β subunit (PobB, CAA55401.1) of 4-carboxyl diphenyl ether dioxygenase from *P. pseudoalcaligenes* POB310, respectively, and their amino acid identities were 96.33% and 99.06%, respectively (Dehmel et al., 1995). Furthermore, as the phylogenetic tree shows, Sca and Scb were the closest relatives to PobA and PobB, respectively (Supplementary Figure 11), and these proteins function as PobAB to break diphenyl ether bonds (Dehmel et al., 1995).

The dioxygenase gene was cloned with DNA as a template, and a single bright band was generated (Supplementary Figure 12A). Then, the PCR products were cloned and inserted into the pET28a(+) plasmid and expressed in *E. coli* BL21 (DE3). The results showed that the gene was successfully cloned and inserted into pET28a(+), and approximately 1,200 bp (*sca*) and 1,000 bp (*scb*) bands were observed by agarose gel electrophoresis (Supplementary Figures 12B, C).

The engineered bacteria were designated *E. coli* BL21(DE3)-pET-28a(+)-*sca* (SCA) and *E. coli* BL21(DE3)-pET-28a(+)-*scb* (SCB), corresponding to *sca* and *scb*, respectively. The 20 mg/L 3-PBA was almost completely degraded by SCA within 24 h, while only 0.53% SCB degraded after 48 h (Supplementary Figure 12D). Therefore, *sca* can be considered a crucial gene for 3-PBA degradation, as SCB had no effect on 3-PBA degradation.

Bioinformatics analysis was performed based on the whole-genome sequence and gene annotation results of strain SC-1, cloning and validation of dioxygenase genes were performed, and a crucial 3-PBA-degrading gene, *sca*, was identified. The length of *sca* is 1,230 bp, the *sca* gene encodes 409 amino acids, and the molecular mass of Sca is approximately 46.3 kDa. The percentages of α-helices, β-folds, and irregular curls in the secondary structure were 20.54%, 5.38%, and 54.03%, respectively (Supplementary Figure 13). Sca has typical Rieske non-heme iron oxygenase (RO) family and phthalate 4,5-dioxygenase (PhDO) subfamily domains (Supplementary Figure 14).

### 3.3 Assessment of 3-PBA degradation activity across distinct cellular states

**FIGURE 1:**
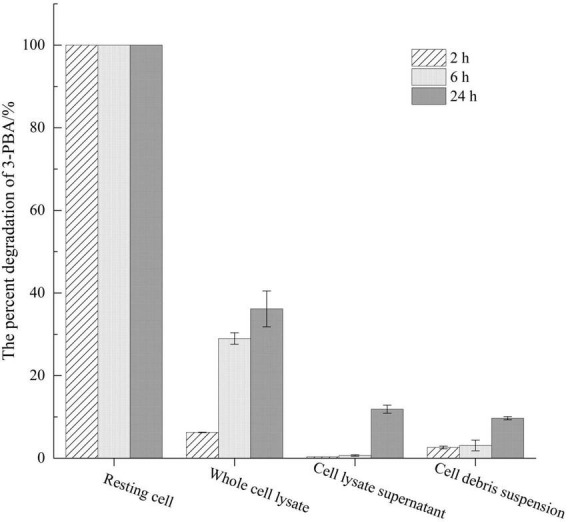
Assessment of 3-PBA degradation activity across distinct cellular states.

The degradation of 3-PBA was detected using resting cells of genetically engineered bacteria and a recombinant crude enzyme solution (Figure 1). After the resting cells were induced with IPTG for 16 h, the cells were obtained through a 0.1 mol/L PBS wash, and these cells could completely degrade 20 mg/L 3-PBA within 2 h. The percent degradation of 3-PBA in the whole-cell lysate of resting *sca* engineered bacteria, at an equivalent cell concentration, following ultrasonic disruption was only 6.25% after 2 h. Furthermore, the percent degradation of 3-PBA gradually increased with prolonged reaction time, reaching 28.98% and 36.17% at 6 and 24 h, respectively. The percent degradation of 3-PBA in the cell fragmentation supernatant and cell debris suspension obtained by centrifugation was less than 3% at 2 and 6 h, respectively. When the reaction time was extended to 24 h, the percent degradation increased to 11.88% and 9.69%, respectively. The percent degradation of 3-PBA in intact resting cells was significantly greater than that in whole-cell lysates, cell lysate supernatants, and cellular debris suspensions following ultrasonic disruption.

### 3.4 Characterizing 3-PBA degradation in resting cells

#### 3.4.1 Time-course degradation and metabolite identification of 3-PBA in resting cells

**FIGURE 2:**
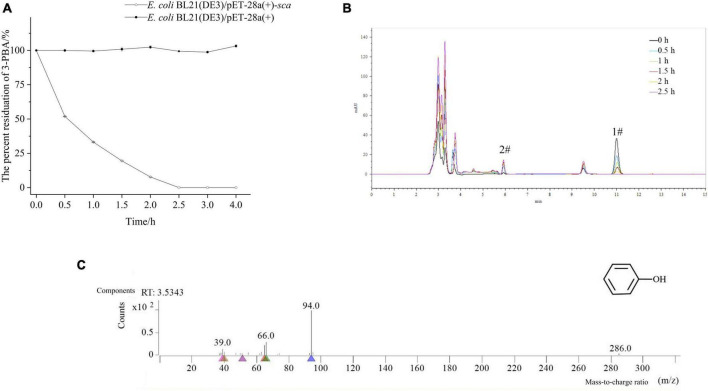
Time-course degradation and metabolite identification of 3-PBA in resting cells. **(A)** Time-course degradation of 3-PBA in resting cells. **(B)** Characterization of 3-PBA degradation by the resting cell process of *sca*-engineered bacteria via HPLC. 1#, 3-PBA; #2, the term degradation product. **(C)** The mass spectra (GC-MS) of 3-PBA degradation by resting cells of *sca*-engineered bacteria.

The process of 3-PBA degradation in resting cells is shown in Figure 2A. The resting cells exhibited a degradation capacity of approximately 50% for 3-PBA within 0.5 h, and complete degradation of 3-PBA was achieved within 2.5 h, demonstrating the robust degradation ability of 3-PBA. The degradation products were analyzed, as shown in Figures 2B, C and Supplementary Figure 15. The peak area of 3-PBA (#1) gradually decreased with time, while a new substance (#2) was generated (Figure 2B). Based on preliminary HPLC analysis, the degradation products of 3-PBA may be phenol (#2). The degradation of 3-PBA products by resting cells was further analyzed using GC-MS, and the resulting mass spectrum is presented in Figure 2C. The NIST database was utilized for GC-MS analysis to identify the total ion flow, and the material with an ion peak at m/z 94 was successfully matched, leading to the identification of one degradation product as phenol.

#### 3.4.2 The optimum temperature and pH

To investigate the optimal reaction temperature for 3-PBA degradation in resting cells, the percent degradation of 3-PBA was measured at various temperatures. With increasing temperature, the resting cells completely reacted with the substrate 3-PBA, leading to a gradual increase in the percent degradation of 3-PBA. At 35°C, 3-PBA was almost completely degraded, and the percent degradation exceeded 50% within 30–45°C. When the reaction temperature exceeded 35°C, the percent degradation of 3-PBA gradually decreased with increasing temperature. At a reaction temperature of 55°C, the degradation of 3-PBA by the resting cells was negligible. An increase in temperature likely triggers cellular apoptosis, ultimately resulting in a decrease or cessation of enzymatic activity.

**FIGURE 3:**
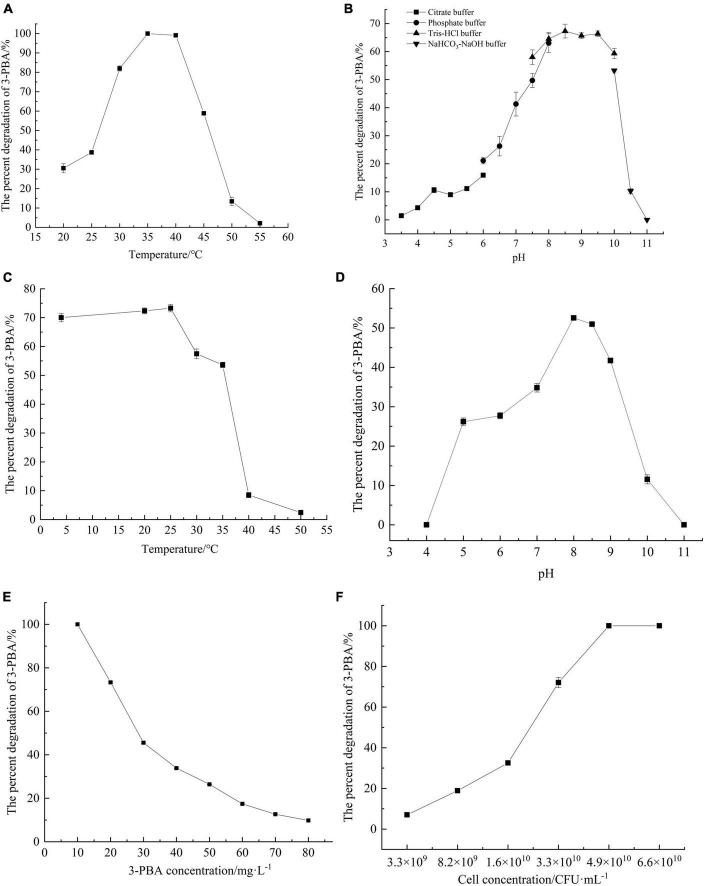
Characterization of 3-PBA degradation by resting cells of *sca*-engineered bacteria. **(A)** The optimum temperature. **(B)** The optimum pH. **(C)** The temperature stability. **(D)** pH stability. **(E)** The substrate tolerance concentrations. **(F)** The impact of cell concentration on 3-PBA degradation.

The percent degradation of 3-PBA by the resting cells under various pH conditions was investigated (Figures 3A, B). The optimal pH for 3-PBA degradation by the resting cells was 8.5, resulting in a percent degradation of 67.3%. The percent degradation of 3-PBA was significantly greater than 40% at pH values ranging from 7.0 to 10.0, indicating that the enzyme is robust at degrading 3-PBA under neutral or alkaline conditions. However, at pH values below 6.0, the percent degradation of 3-PBA decreased to less than 20%, while a sharp decrease in percent degradation occurred when the pH exceeded 10.0. The deviation from the optimal pH due to overacidic or overalkaline conditions may adversely affect cellular viability, leading to cell death and altering the dissociation state of enzyme active groups; these effects lead to irreversible enzyme degeneration and loss of 3-PBA degradation activity.

#### 3.4.3 Temperature and pH stability

The percent degradation of 3-PBA in resting cells remained above 50% even after the cells were exposed to temperatures between 4 and 35°C for 1 h, as depicted in Figures 3C, D, indicating remarkable stability within this temperature range. When the temperature exceeded 25°C, the percent degradation of 3-PBA gradually decreased with increasing holding temperature. Following a 1-h incubation at 40°C, the percent degradation of 3-PBA in quiescent cells decreased to approximately 8%. The enzyme activity significantly deteriorated at or above 40°C.

In terms of pH stability, the percent degradation of 3-PBA in resting cells significantly decreased (to less than 10%) following a 1-h incubation at pH values below 5.0 or above 9.0 at a constant temperature of 35°C. The percent degradation of 3-PBA exceeded 25% within 1 h in the buffer solution at 35°C and pH values between 5.0 and 9.0, indicating that the enzyme tolerates this pH range.

#### 3.4.4 The effect of metal ions

The impact of metal ions on the degradation of 3-PBA in resting cells is presented in Table 1. Compared to that of the control group without metal ions, the percent degradation of 3-PBA was significantly reduced in the presence of 1 mmol/L Cu^2+^, Mn^2+^, Zn^2+^, and Cd^2+^, which effectively inhibited the degradation of 3-PBA in resting cells. However, at a concentration of 1 mmol/L, Na^+^, K^+^, Ca^2+^, Mg^2+^, Pb^2+^, Fe^2+^, Fe^3+^, and Ba^2+^ did not have any significant effect on the percent degradation of 3-PBA, while Pb^2+^ and Fe^2+^ at a concentration of 5 mmol/L significantly inhibited its percent degradation.

**TABLE 1:** The impact of metal ions on the degradation of 3-PBA in resting cells.

Metal ion	The rate of 3-PBA degradation/%	
	**1 mmol/L**	**5 mmol/L**
Control	54.80 ± 3.85^a^	54.80 ± 3.85^ab^
Na^+^	54.97 ± 0.01^a^	55.48 ± 1.00^ab^
K^+^	54.92 ± 0.05^a^	58.54 ± 2.78^a^
Ca^2+^	54.53 ± 0.12^a^	50.57 ± 2.96^b^
Cu^2+^	5.65 ± 0.41^cd^	–
Mg^2+^	54.98 ± 0.00^a^	58.57 ± 2.52^a^
Mn^2+^	35.40 ± 0.36^b^	–
Pb^2+^	54.97 ± 0.02^a^	21.61 ± 2.96^d^
Zn^2+^	7.44 ± 0.25^c^	–
Fe^2+^	54.98 ± 0.00^a^	38.53 ± 1.25^c^
Fe^3+^	54.98 ± 0.01^a^	51.25 ± 2.33^b^
Cd^2+^	3.93 ± 0.14^d^	–
Ba^2+^	54.98 ± 0.00^a^	56.62 ± 2.04^ab^

The identical lowercase letters in the same column denote negligible differences (*P* > 0.05), and the employment of distinct lowercase letters signifies notable differences (*P* < 0.05).

#### 3.4.5 The effects of chemical reagents

The percent degradation of 3-PBA was determined by adding chemical reagents at varying concentrations to the degradation reaction system. The results are presented in Tables 2, 3. Compared to the control group, the presence of 1 mmol/L SDS, CTAB, 1% ethyl alcohol, isopropyl alcohol, acetone, and n-hexane resulted in a significant decrease in 3-PBA in the resting cells. The percent degradation of 3-PBA was not significantly affected by the presence of 1 mmol/L DTT, EDTA, urea, 1% methanol, polyethylene glycol, or dimethyl sulfoxide. However, 3-PBA was inhibited when 5% methanol, polyethylene glycol, ethyl alcohol, and dimethyl sulfoxide were added.

**TABLE 2:** The impact of chemical reagents on the degradation of 3-PBA in resting cells.

Chemical reagent	The rate of 3-PBA degradation/%	
	**1 mmol/L**	**5 mmol/L**
Control	54.80 ± 3.85^a^	54.80 ± 3.85^a^
SDS	39.49 ± 0.48^b^	–
DTT	54.04 ± 0.81^a^	53.98 ± 0.30^a^
CTAB	31.66 ± 0.71^c^	–
EDTA	54.50 ± 0.28^a^	55.82 ± 2.76^a^
Urea	54.79 ± 0.06^a^	57.94 ± 0.74^a^

The identical lowercase letters in the same column denote negligible differences (*P* > 0.05), and the employment of distinct lowercase letters signifies notable differences (*P* < 0.05).

**TABLE 3:** The impact of organic solvents on the degradation of 3-PBA in resting cells.

Organic solvent	The rate of 3-PBA degradation/%	
	**1%**	**5%**
Control	54.80 ± 3.85^a^	54.80 ± 3.85^ab^
Methanol	54.63 ± 0.23^ab^	45.51 ± 0.57^c^
Ethyl alcohol	51.61 ± 0.58^ab^	34.52 ± 0.03^d^
Isopropyl alcohol	34.27 ± 0.93^d^	–
Polyethylene glycol	54.30 ± 0.20^ab^	49.98 ± 1.19^bc^
Acetone	38.67 ± 0.91^c^	–
Dimethyl sulfoxide	54.78 ± 0.06^a^	47.32 ± 0.31^c^
N-hexane	50.82 ± 0.10^b^	57.39 ± 2.32^a^

The identical lowercase letters in the same column denote negligible differences (*P* > 0.05), and the employment of distinct lowercase letters signifies notable differences (*P* < 0.05).

#### 3.4.6 Determination of substrate tolerance

Varying concentrations of 3-PBA were added to the conversion reaction system to investigate the substrate tolerance of the resting cells (Figure 3E). When the concentration of 3-PBA ranged from 10 to 80 mg/L, the degradation capability of all resting cells was proficient. Moreover, at a concentration of 10 mg/L, 3-PBA was completely degraded. With increasing 3-PBA concentration, the percent degradation gradually decreased until a steady state was reached. A high concentration of 3-PBA may cause cytotoxicity in cells.

#### 3.4.7 The effect of cell concentration

The impact of the resting cell concentration on the degradation of 3-PBA was investigated (Figure 3F). At a cell concentration of 3.3 × 10^9^CFU/ml, the percent degradation of 3-PBA was only 7%. As the resting cell concentration increased, the percent degradation gradually increased. At a concentration of 4.9 × 10^10^ CFU/ml, nearly complete degradation of 3-PBA was achieved. The higher the cell concentration was, the greater the enzyme content in the reaction system, leading to an increase in the percent degradation of 3-PBA.

#### 3.4.8 Substrate specificity determination

The degradation of various substrates by quiescent cells was investigated, as depicted in Supplementary Table 10. Resting cells degraded 4′-HO-3-PBA and 3′-HO-4-PBA but did not degrade the monophenyl compounds that were possibly generated during the degradation of 3-PBA. The structures of HO-3-PBA and 3-PBA are similar and both feature diphenyl ether bonds, suggesting that the enzymatic function of the *sca* protein is to break these bonds.

## 4 Discussion

The primary metabolite in the biodegradation pathway of PYRs is 3-PBA, which is extensively detected in food, human urine, and the environment. In this study, we performed whole-genome sequencing of *Sphingomonas baderi* SC-1 and identified the vital 3-PBA-degrading gene *sca* (1230 bp). After bacteria were engineered to express *sca*, a concentration of 20 mg/L 3-PBA was completely degraded within 24 h, yielding phenol as one of degradation products. This finding corresponds with the annotation of *sca* as a 3-PBA dioxygenase based on whole-genome sequencing of *Sphingomonas baderi* SC-1, indicating that the experimental results and genomic evidence are consistent. Previous studies have established that ortho-dioxygenases are Rieske non-heme iron enzymes involved in the hydroxylation of aromatic rings (known as ring-hydroxylating oxygenases, RHOs) (Chakraborty et al., 2012). The subjects were categorized into five distinct classifications (Kweon et al., 2008). The 3-PBA-3,4-angular dioxygenase PobAB from *P. pseudoalcaligenes* POB310 is a type I RHO with two open reading frames (ORFs), *pobA* and *pobB*, which encode oxygenase and FNRC-type reductase subunits, respectively. Except for the presence of FNRN-type reductase, type II RHOs exhibit similarities to Type I RHOs. The Type III variant is characterized by the presence of an FNRN-type reductase and the absence of a [2Fe-2S]-type ferredoxin subunit. 3-PBA-1′,2′-Angular dioxygenase, known as PbaA1A2BC from *S. wenxiniae* JZ-1, is a component of Type IV RHOs and comprises the following ORFs: *pbaA1*, *pbaA2*, *pbaB*, and *pbaC*. Among these, *pbaA* and *pbaB* are responsible for the oxygenase activity and [2Fe-2S]-type ferredoxin subunit, respectively.

Additionally, the coexpression of *pbaAB* with *pbaC*, which corresponds to glutathione reductase (GR), is needed. The Type V system incorporates a distinct [3Fe-4S]-type ferredoxin, which distinguishes it from Type IV. In general, the reduction equivalents necessary for RHO-related catalytic reactions are provided by NAD(P)H-dependent reductase proteins through the electron transport chain. However, this approach involves a significant challenge, as *in vitro* activity is insufficient. Few efficient methods have been proposed for the screening and identification of 3-PBA oxygenase due to its unique characteristics; 3-PBA oxygenase primarily occurs as membrane proteins or multisubunit proteins, imposing limitations on its separation and purification (Zhao et al., 2021). Currently, two 3-PBA ortho-dioxygenase genes, namely, *pobAB* (Dehmel et al., 1995) and *pbaA1A2B* (Wang et al., 2014), have been successfully cloned. Both genes were obtained using deletion mutants and subsequently confirmed via genetic recall, employing different aromatic rings. Among them, PobAB enzymatically catalyzes the conversion of the carboxyl aromatic ring of 3-PBA to phenol and protocatechuic acid, which corresponds with the findings of this study. This process involves the introduction of two hydroxyl groups at the 3- and 4-positions of the benzoic acid moiety, followed by cleavage of the diphenyl ether bond, which is facilitated by ortho-dioxygenase (Zhao et al., 2021). The enzyme PbaA1A2BC facilitates the conversion of the non-carboxyl aromatic ring to 3-hydroxybenzoic acid and catechol, aligning with the pathway that involves 3-PBA metabolism by *S. wenxiniae* JZ-1. These findings suggest that ortho-dioxygenase cleaves the diphenyl ether bond at positions 1′ and 2′ of the aromatic moiety (Zhao et al., 2021). The degradation of diphenyl ether structure analogs may be associated with lignin oxidase and cytochrome P450 monooxygenases (CYPs) (White et al., 1996). Lignin oxidase primarily comprises lignin peroxidase, manganese peroxidase, and laccase. Filamentous fungi, which are abundant in these enzymes, serve as the predominant microbial degraders of diphenyl ether derivatives (Dashtban et al., 2009). CYPs, which are widely distributed across all biological kingdoms, are thiolate heme proteins that primarily function as terminal monooxygenases in a variety of biochemical reactions, such as hydroxylation, by catalyzing diverse oxidative transformations of various molecules (Nelson, 2009). After determining the enzymatic activity of CYP, Zhao et al. (2020) concluded that CYP played a significant role in cleaving the 3-PBA diphenyl ether bond by *Aspergillus oryzae* M-4; thus, CYP facilitates the complete degradation of downstream products through synergistic action with laccase and lignin peroxidase. Transcriptome analysis of *A. oryzae* M-4 also revealed that 3-PBA induced the upregulation of genes associated with energy expenditure, the oxidative stress response, membrane transport, and DNA repair. In-depth functional interpretation of differentially expressed genes suggested that the involvement of CYP450 may contribute to the hydroxylation of 3-PBA (Hu et al., 2023).

Additionally, we observed a fascinating phenomenon: the percentage degradation of 3-PBA in the cellular lysate of the *sca*-engineered bacteria was exceptionally low. Notably, maintaining complete cellular integrity was crucial for the efficient degradation of 3-PBA. This relatively distinctive phenomenon was scrutinized via subsequent analysis in the current investigation. The Sca protein expressed in prokaryotes is presumably a membrane protein. The adsorption and entry of Sca into cells are aided by the presence of living cells, while its degradation depends on the integrity of membrane proteins. However, after cells were disrupted by ultrasound, the exposure of membrane proteins to a hydrophilic environment promoted the aggregation of proteins in the cell debris suspension due to their hydrophobic nature, leading to their inactivation (Zhang et al., 2021). The CellPLoc package was utilized to predict the subcellular localization of the dioxygenase protein Sca, which is encoded by the 3-PBA-degrading gene *sca*. This protein predominantly localizes to the cell membrane. Another plausible hypothesis is that Sca functions as a dioxygenase belonging to the oxidoreductase class and aids in the degradation of 3-PBA by utilizing coenzymes, cofactors, and cogroups for efficient electron provision and transfer (Zhao et al., 2020, 2022). This finding highlights a promising avenue for future investigations, in which the dioxygenase gene from *Sphingomonas baderi* was successfully isolated and characterized; in addition, the results revealed the potential of this gene for efficient degradation of 3-PBA.

## 5 Conclusion

The crucial 3-PBA-degrading gene *sca* (1,230 bp) was identified through whole-genome sequencing of *Sphingomonas* sp. SC-1. The degradation properties of 3-PBA were investigated using resting cells of genetically engineered bacteria harboring the *sca* gene. Phenol was identified as one of degradation byproducts. In addition to their ability to degrade 3-PBA, these resting cells demonstrated proficiency in degrading 4′-HO-3-PBA and 3′-HO-4-PBA.

## Data availability statement

The datasets presented in this study can be found in online repositories. The names of the repository/repositories and accession number(s) can be found below: NCBI – PRJNA981288.

## Author contributions

QL: Conceptualization, Formal analysis, Supervision, Writing – original draft. QZ: Data curation, Formal analysis, Investigation, Writing – original draft. YC: Data curation, Formal analysis, Investigation, Writing – original draft. KH: Formal analysis, Investigation, Methodology, Writing – review & editing. MS: Formal analysis, Investigation, Methodology, Writing – review & editing. JL: Formal analysis, Methodology, Writing – review & editing. AL: Formal analysis, Methodology, Writing – review & editing. LZ: Methodology, Resources, Writing – review & editing. SL: Conceptualization, Funding acquisition, Project administration, Supervision, Writing – review & editing.

## Footnotes

1
www.majorbio.com
