# Supplementary material for: Whole-genome sequencing of Sphingobium baderi SC-1 and identification of a crucial 3-phenoxybenzoic acid-degrading gene
Source: Front Microbiol. 2024 Apr 5;15:1361335. doi: 10.3389/fmicb.2024.1361335 (PMC11026547; doi:10.3389/fmicb.2024.1361335)
Supplement: Supplementary file 1 [file Data_Sheet_1.DOCX]

**Frontiers in Microbiology**

**Supplementary material for**

Whole genome sequencing of *Sphingobium baderi* SC-1 and identification of a crucial 3-phenoxybenzoic acid-degrading gene

Qin Li^a, 1^, Qiao Zhou^a, 1^, Yuan Chen^a, 1^, Kaidi Hu^a^, Montserrat Sarrà^b^, Jianlong Li^a^, Aiping Liu^a^, Likou Zou^c^, Shuliang Liu^a,^ *

^a^College of Food Science, Sichuan Agricultural University, Ya’an, Sichuan 625014, People’s Republic of China

^b^Departament d’Enginyeria Química, Biològica i Ambiental, Escola d’Enginyeria, Universitat Autònoma de Barcelona, 08193, Bellaterra, Barcelona, Spain

^c^College of Resources, Sichuan Agricultural University, Chengdu, Sichuan 611130, People’s Republic of China

^1^Qin Li, Qiao Zhou and Yuan Chen are contributed equally to the work.

*Corresponding author at: College of Food Science, Sichuan Agricultural University, Ya’an, Sichuan 625014, People’s Republic of China.

E-mail address: lsliang999@163.com (S. Liu).

Table S1 The primer sequence of target gene of strain SC-1

| Gene | Primer | Primer sequence（5’-3’） |
| --- | --- | --- |
| *sca* | *sca*-F | ATGAGCAAGACCATTCCCATCGTCG |
|  | *sca*-R | TCAGGCCGCGTCGGTTTCGGTCTT |
| *scb* | *scb*-F | GTGAGCGCCGCAGCGACCATGGC |
|  | *scb*-R | CTAGAGGTCCAGCGTCAGCTCCGCC |


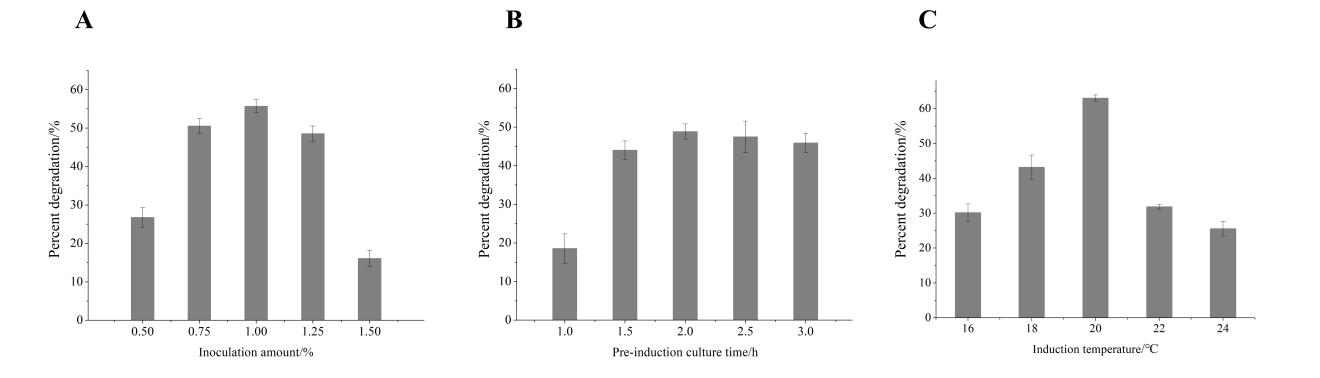


Fig. S1 Effect of inoculum volume (A), pre-induction culture time (B) and induction temperature (C) on the percent degradation of 3-PBA

Table S2 Factor and level of Box-Behnken design

| Level | A Preinduction culture time/h | B Inoculation amount/% | C Induction temperature/℃ |
| --- | --- | --- | --- |
| -1 | 1.5 | 0.5 | 18 |
| 0 | 2.0 | 1.0 | 20 |
| 1 | 2.5 | 1.5 | 22 |

Table S3 Experiment design and result of 3-PBA- degradation by SCA

| Number | A | B | C | Percent degradation/% |
| --- | --- | --- | --- | --- |
| 1 | 0 | 1 | 1 | 43.95 |
| 2 | 1 | 0 | -1 | 43.62 |
| 3 | -1 | -1 | 0 | 11.53 |
| 4 | 1 | 0 | 1 | 53.89 |
| 5 | 0 | 0 | 0 | 71.65 |
| 6 | 0 | 0 | 0 | 65.05 |
| 7 | 0 | 0 | 0 | 59.18 |
| 8 | 1 | 1 | 0 | 76.07 |
| 9 | 0 | 0 | 0 | 52.27 |
| 10 | 0 | 1 | -1 | 55.76 |
| 11 | 0 | 0 | 0 | 66.15 |
| 12 | -1 | 0 | 1 | 28.12 |
| 13 | -1 | 1 | 0 | 29.26 |
| 14 | -1 | 0 | -1 | 47.65 |
| 15 | 0 | -1 | -1 | 45.43 |
| 16 | 1 | -1 | 0 | 26.75 |
| 17 | 0 | -1 | 1 | 44.51 |


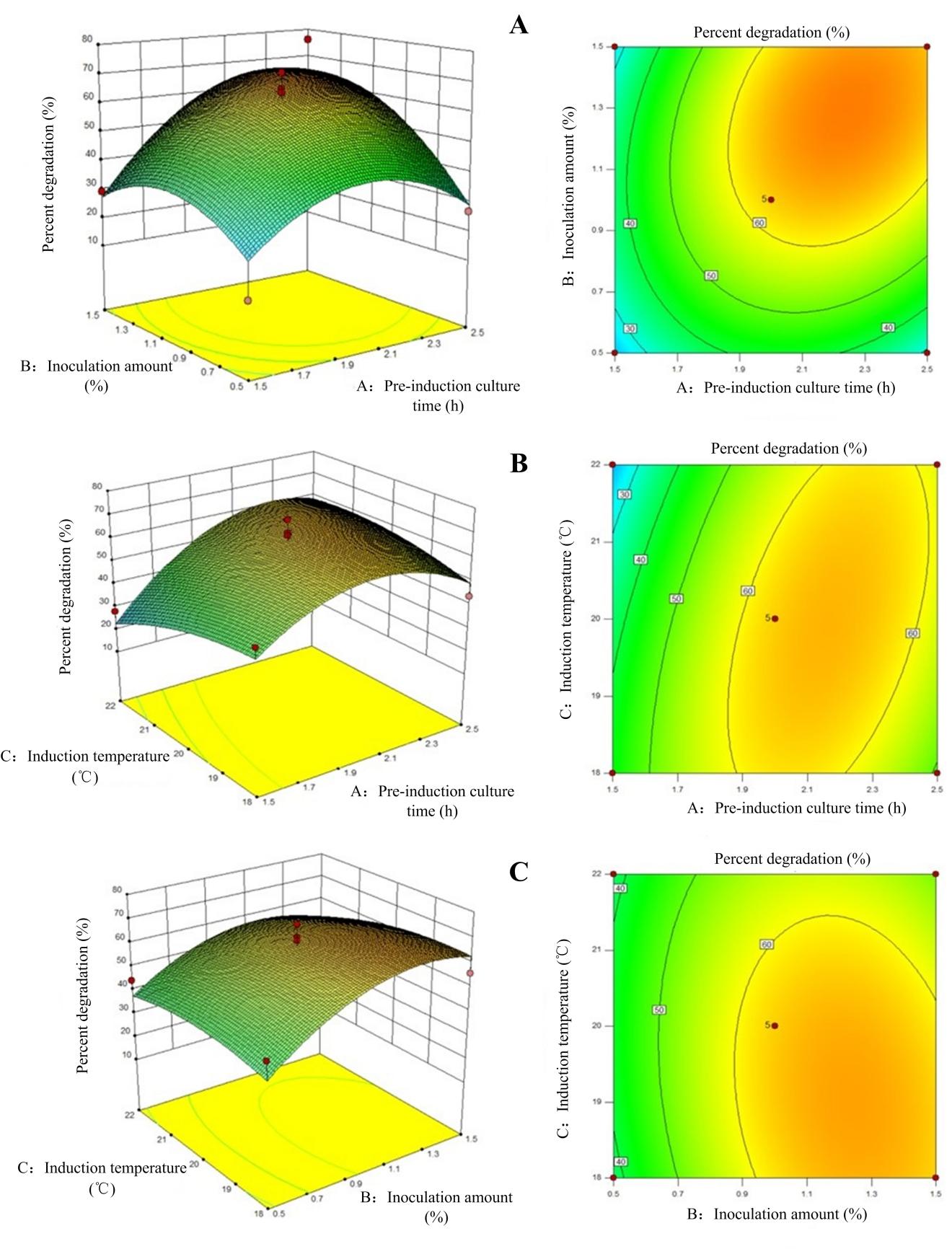


Fig. S2 The stereogram of response surface and contour map

A: inoculation amount and pre-induction culture time; B: induction temperature and pre-induction culture time; C: induction temperature and inoculation amount.


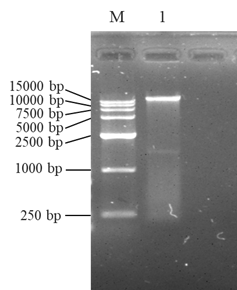


Fig. S3 Electrophoresis of DNA extracted from *Sphingomonas* sp.SC-1

Table S4 The statistics of Illumina sequencing data

| Name | Number | Name | Number |
| --- | --- | --- | --- |
| Read Len (bp) | 150 | Clean Pair Reads | 6918410*2 |
| Raw Pair Reads | 7098202*2 | Clean Single Reads | 143552 |
| Raw bases (bp) | 2143657004 | Clean bases (bp) | 2078299294 |
| Raw Q20 (%) | 97.48 | Clean Q20 (%) | 98.22 |
| Raw Q30 (%) | 93.12 | Clean Q30 (%) | 94.15 |

Note: Read Len, the length of the original reads; Raw Pair reads, the number of double-ended reads of the original sequence data; Raw bases, the total base number of raw data (Raw Pair reads×Read Len); Raw Q20、Q30, the percentage of bases with error identification rates were less than 0.01 and 0.001 in the original data; Clean Pair Reads, the number of double-end reads after quality control; Clean Single Reads, the number of single end reads after quality control; Clean bases, the total number of bases after quality control (Clean Pair Reads×Read Len); Clean Q20、Q30, the percentage of bases with error identification rate were less than 0.01 and 0.001 in the total bases after data quality control.

Fig. S4 Quality control data statistics of Illumina

Note: A, base distribution map; B, base mass distribution map.


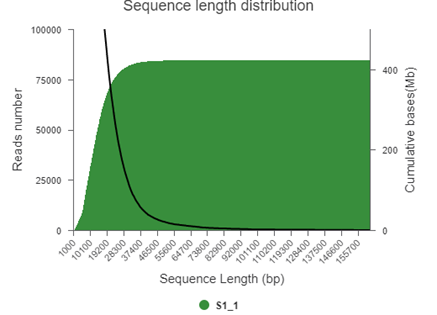


Fig. S5 The statistics of PacBio sequencing reads

Note: Abscissa, the length of sequencing reads; Ordinate, the number of reads of different lengths. The black curve represents the change in the number of reads as the length of reads increases.

Table S5 The results of SC-1 genomic assembly

| Name | Length（bp） | GC（%） |
| --- | --- | --- |
| Genome | 4374142 | 63.53 |
| Chromosome | 3601664 | 63.79 |
| P SC-1-A | 349403 | 62.01 |
| p SC-1-B | 167669 | 63.76 |
| p SC-1-C | 97252 | 61.81 |
| p SC-1-D | 77650 | 62.28 |
| p SC-1-E | 65982 | 61.49 |
| p SC-1-F | 12940 | 60.90 |


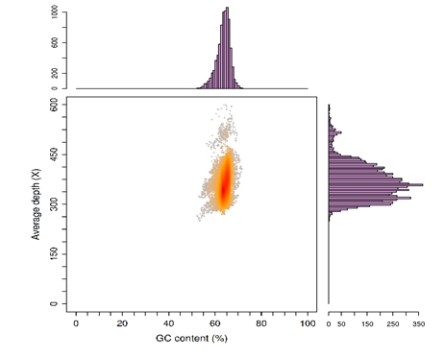


Fig. S6 Average depth distribution of GC content


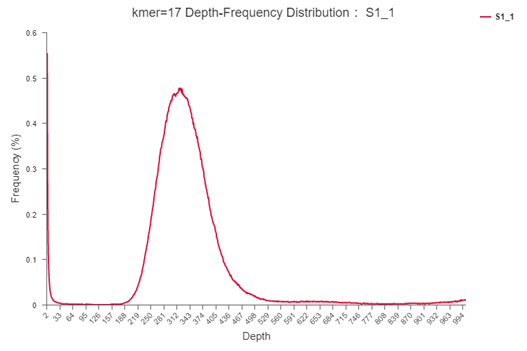


Fig. S7 K-mer frequency distribution curve

Table S6 The gene prediction of SC-1 genome

| Statistical indicators | Value |
| --- | --- |
| Gene number | 4432 |
| Total gene length（bp） | 3890040 |
| Mean gene length（bp） | 877.72 |
| Mean gene density | 1.01 |
| Genes as a percentage of the genome（%） | 88.93 |
| Intergenomic length（bp） | 484102 |
| GC content in intergene region（%） | 58.56 |
| Intergenetic regions as a percentage of the genome（%） | 11.07 |
| The average GC content of genes（%） | 64.15 |

Note: the average gene density is the number of genes per 1kb base in the genome


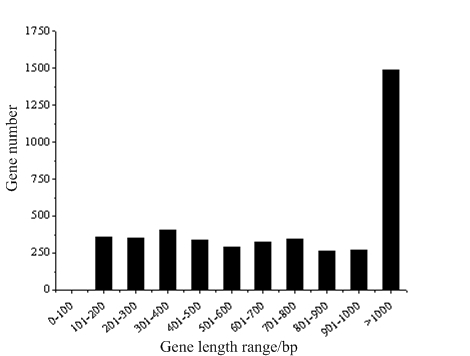


Fig. S8 The distribution of the predicted genes with different length

Table S7 The results of tRNA prediction

| tRNA type | Number | tRNA type | Number | tRNA type | Number | tRNA type | Number | tRNA type | Number |
| --- | --- | --- | --- | --- | --- | --- | --- | --- | --- |
| Ala | 5 | Cys | 1 | His | 1 | Met | 4 | Thr | 3 |
| Arg | 4 | Gln | 2 | Ile | 2 | Phe | 1 | Trp | 1 |
| Asn | 1 | Glu | 2 | Leu | 5 | Pro | 4 | Tyr | 1 |
| Asp | 1 | Gly | 3 | Lys | 3 | Ser | 4 | Val | 3 |

**
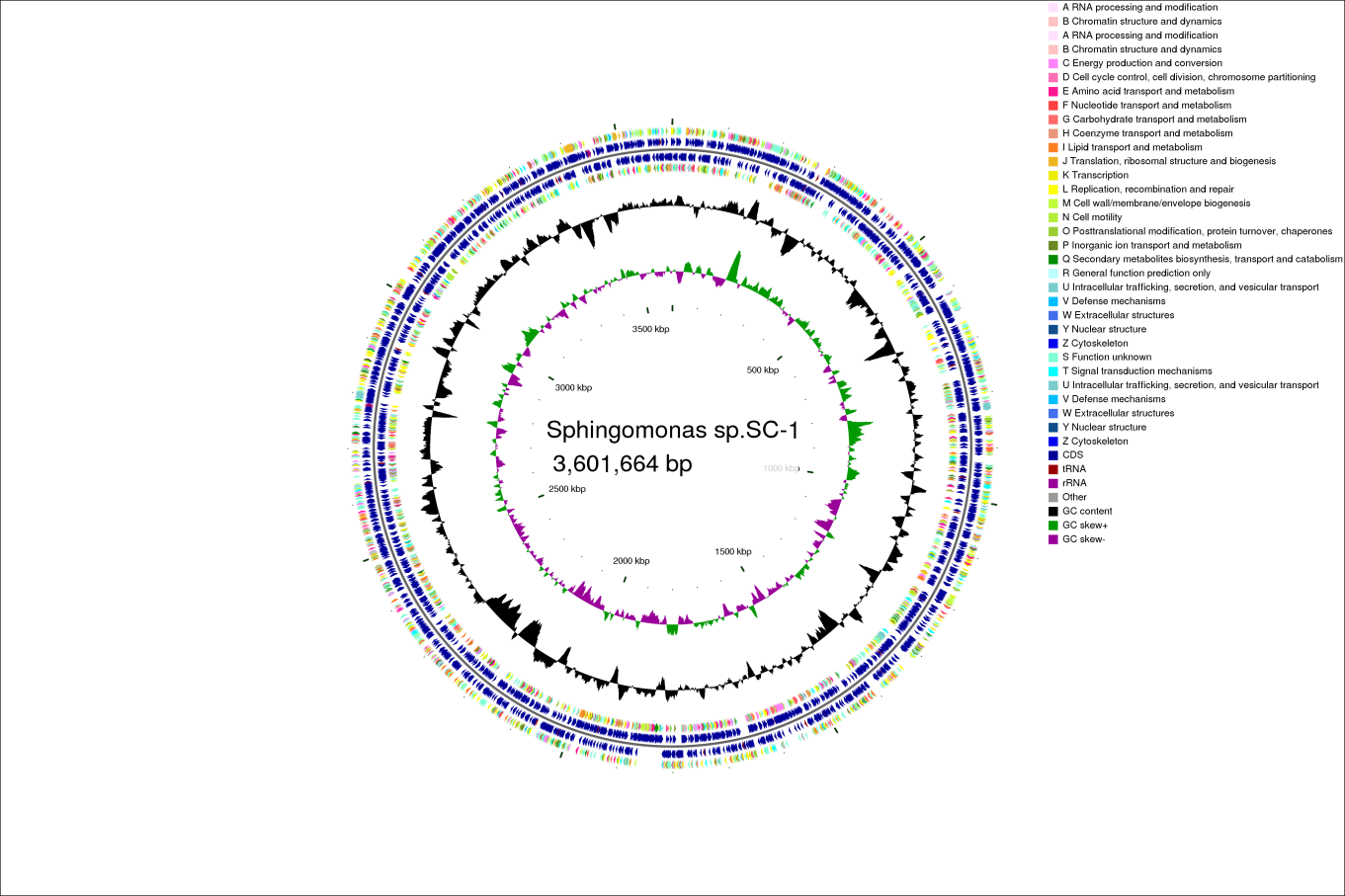
**

a

**
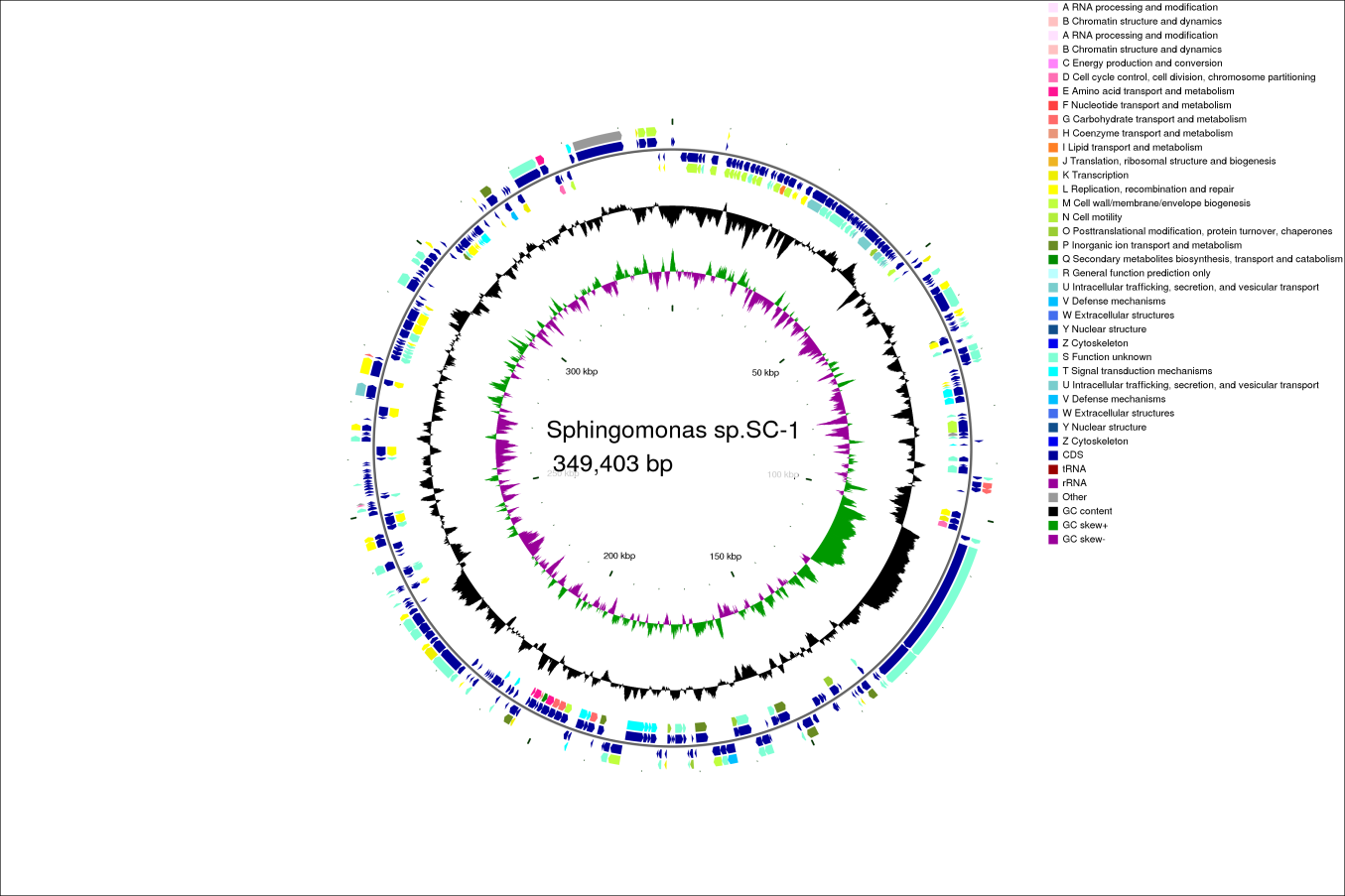
**

b

**
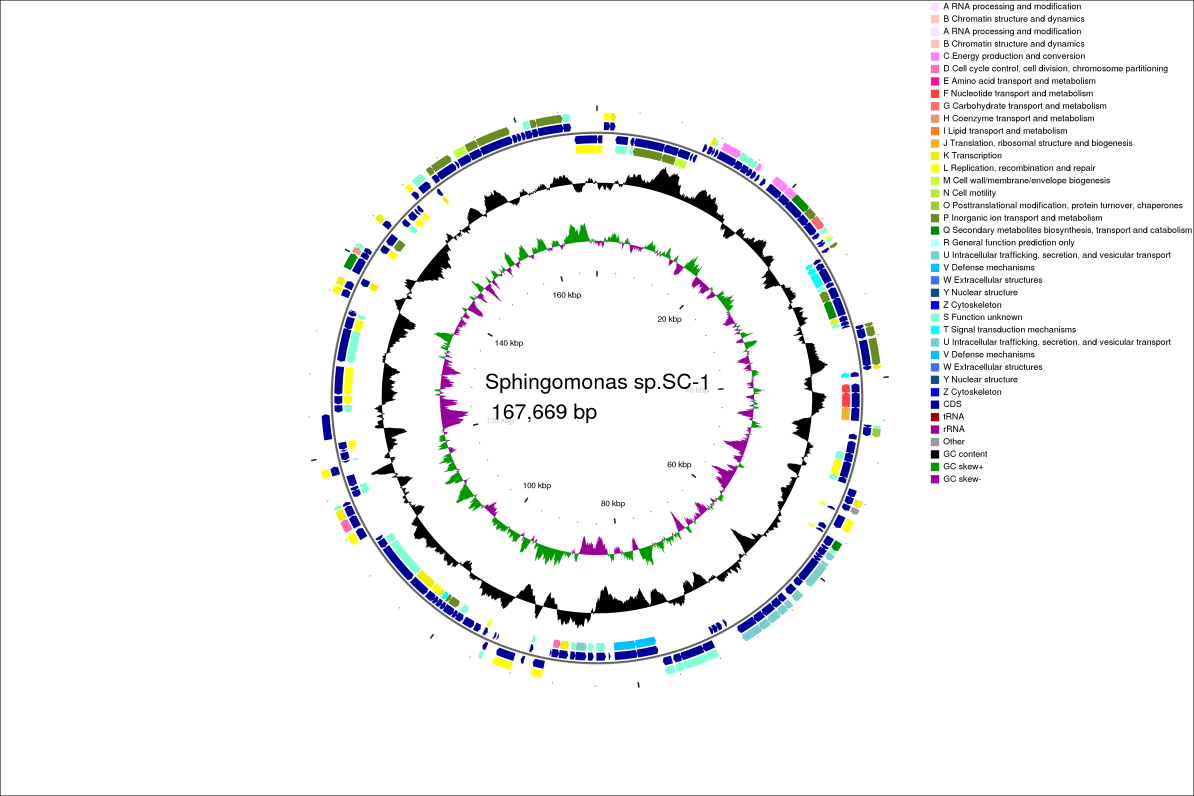
**

c

**
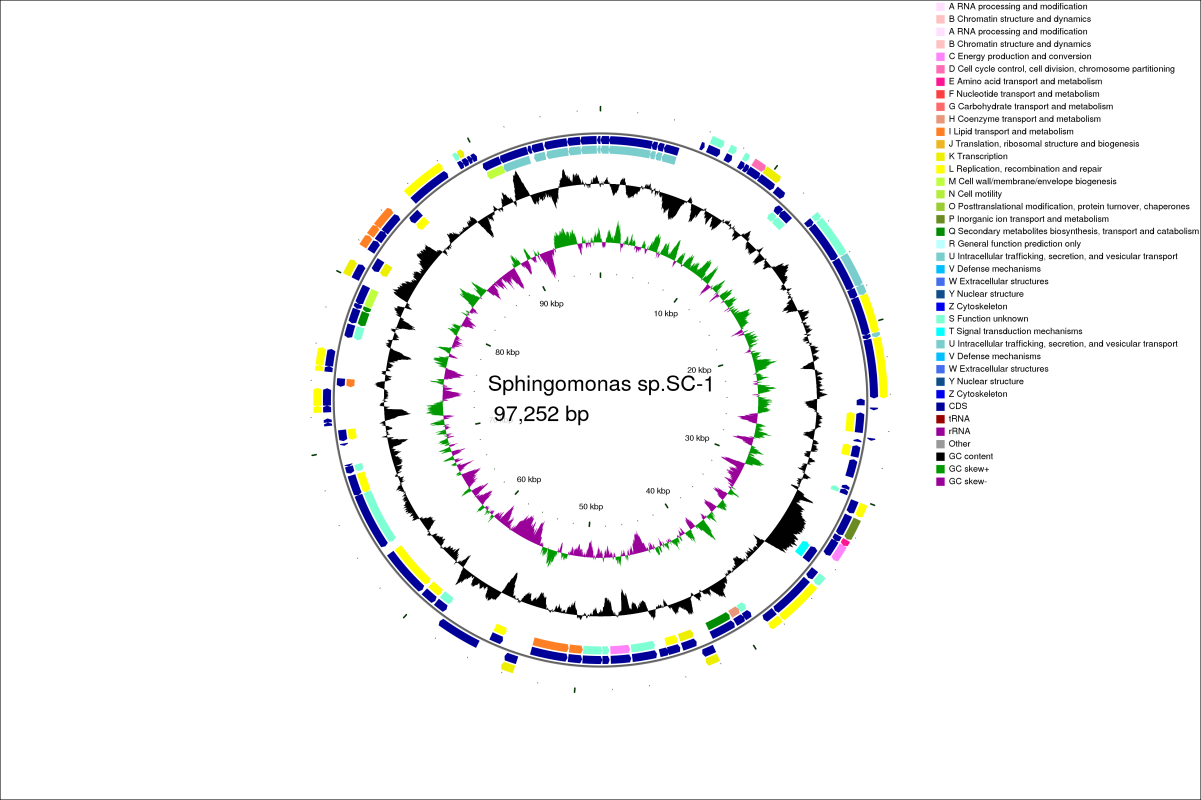
**

d

**
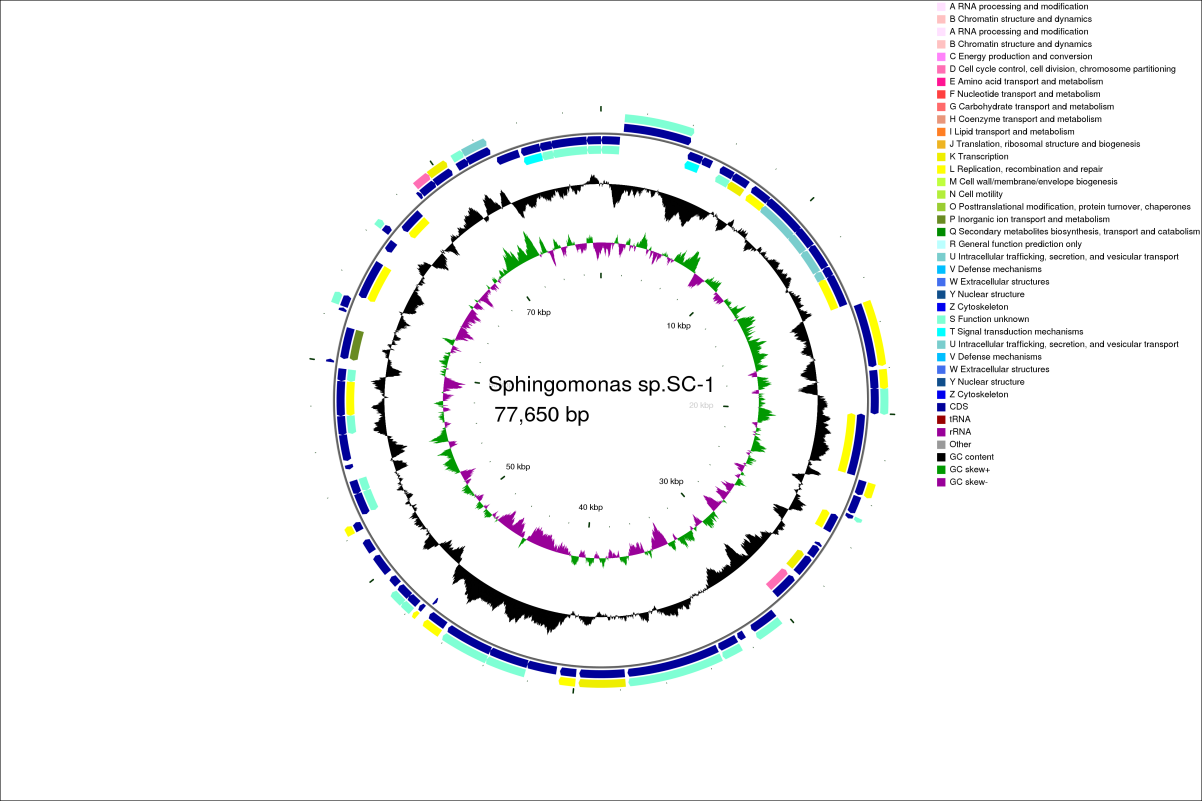
**

e

**
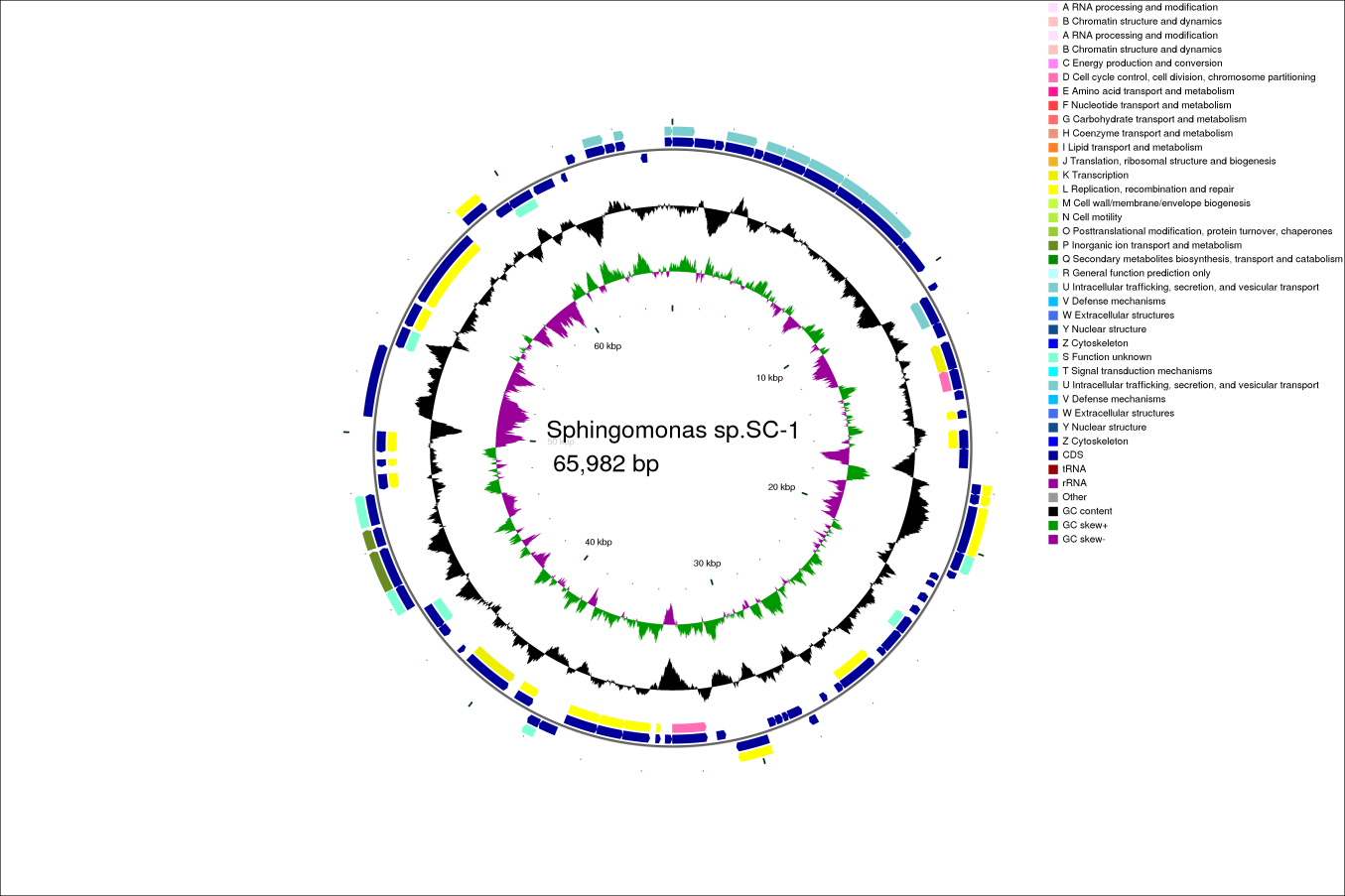
**

f

**
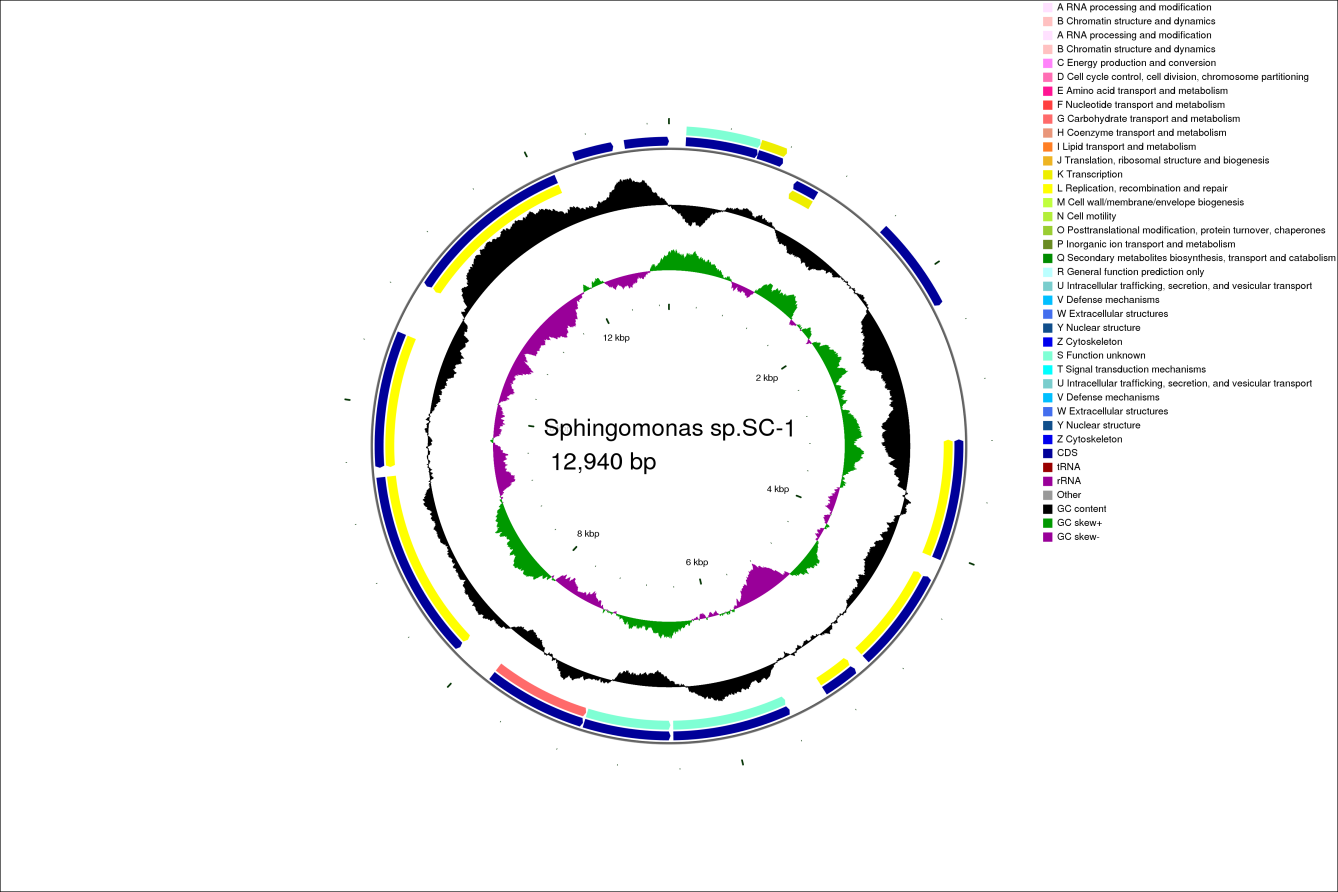
**

g

Fig. S9 The CGView map of SC-1 genome including chromosome and six plasmids; a: chromosome, b: p SC-1-A, c: p SC-1-B, d: p SC-1-C, e: p SC-1-D, f: p SC-1-E, g: p SC-1-F

Genome function annotation:

Genome function annotation was demonstrated mainly through comparison with six biological databases, as shown in Supplementary Data Table S8.

The nonredundant protein database (NR) included 3713, 302, 163, 94, 67, 77 and 16 functional genes annotated on chromosomes and 6 plasmids (pSC-1-A, pSC-1-B, pSC-1-C, pSC-1-D, pSC-1-E, pSC-1-F), respectively. The proportion of predicted functional genes was 100% (4432/4432).

In the Swiss-Prot database, 2436, 143, 106, 68, 29, 40 and 7 functional genes were annotated on chromosomes and on 6 plasmids, respectively. The proportion of predicted functional genes was 63.8% (2829/4432).

In the Pfam database, 2926, 185, 123, 73, 44, 43 and 11 functional genes were annotated on chromosomes and on 6 plasmids, respectively. The proportion of predicted functional genes was 76.8% (3405/4432).

According to the Gene Ontology (GO) database, 2511, 141, 103, 59, 32, 37 and 11 functional genes were annotated on chromosomes and on 6 plasmids, respectively. The proportion of predicted functional genes was 65.3% (2894/4432). The statistics of the GO functional annotations are shown in Fig. S10A.

Clusters of Orthologous Groups of proteins (COG) database: 2888, 204, 129, 76, 50, 43 and 13 functional genes were annotated on chromosomes and on 6 plasmids, respectively. The proportion of predicted functional genes was 76.8% (3403/4432). The COG functional annotation statistics are shown in Fig. S10C.

In the Kyoto Encyclopedia of Genes and Genomes (KEGG) database, 1649, 70, 53, 32, 13, 21 and 3 functional genes were annotated on chromosomes and on 6 plasmids, respectively. The proportion of predicted functional genes was 41.5% (1841/4432). A histogram of the KEGG categories is shown in Fig. S10B.


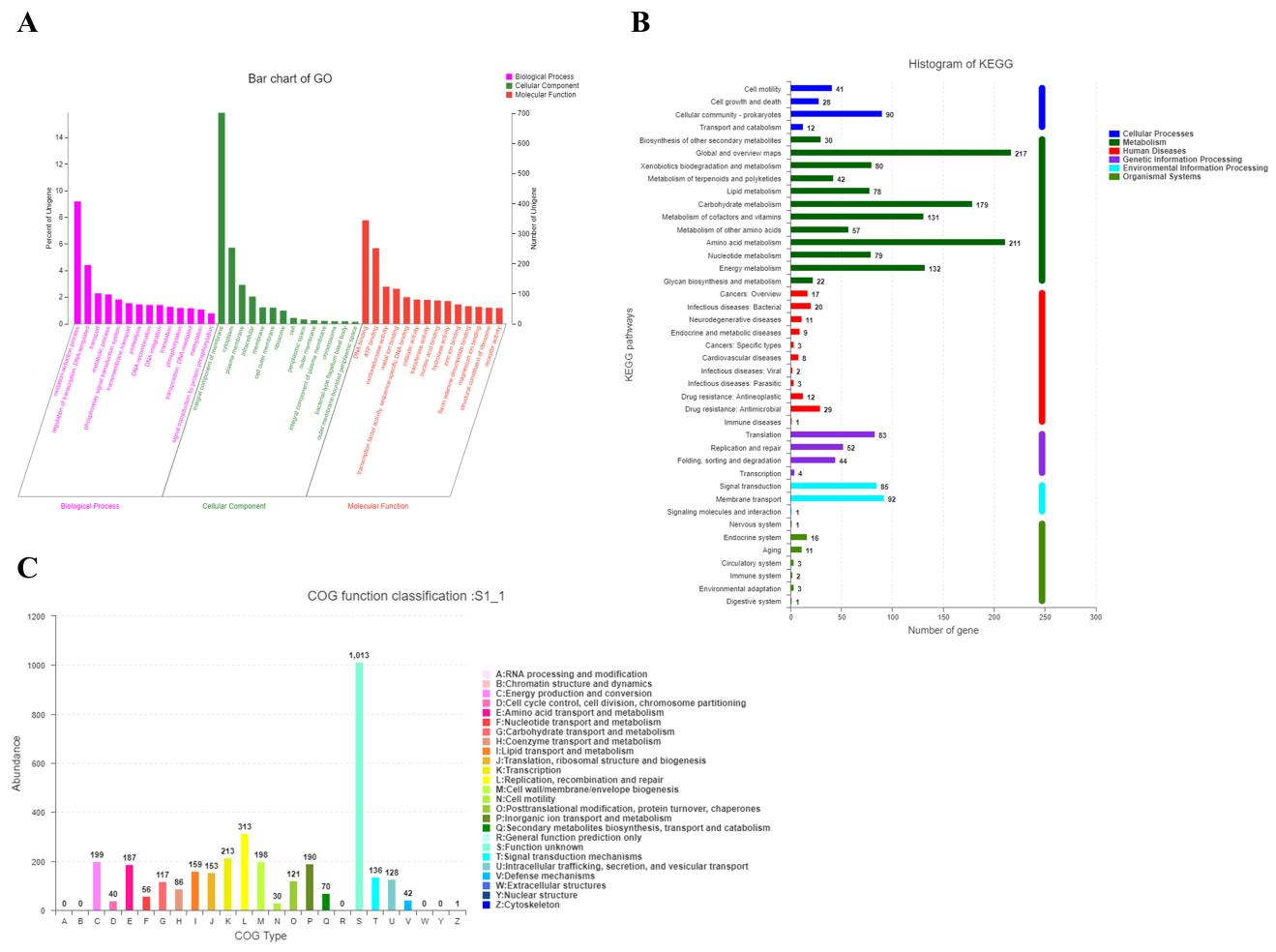


Fig. S10. The statistics of GO (A), KEGG (B) and COG (C) functional annotation

Table S8 The statistics of gene functional annotation

| Genome | NR | Swiss-Prot | Pfam | COG | GO | KEGG |
| --- | --- | --- | --- | --- | --- | --- |
| Chromosome | 3713 | 2436 | 2926 | 2888 | 2511 | 1649 |
| pSC-1-A | 302 | 143 | 185 | 204 | 141 | 70 |
| pSC-1-B | 163 | 106 | 123 | 129 | 103 | 53 |
| pSC-1-C | 94 | 68 | 73 | 76 | 59 | 32 |
| pSC-1-D | 67 | 29 | 44 | 50 | 32 | 13 |
| pSC-1-E | 77 | 40 | 43 | 43 | 37 | 21 |
| pSC-1-F | 16 | 7 | 11 | 13 | 11 | 3 |
| Comment proportion/% | 100 | 63.8 | 76.8 | 76.8 | 65.3 | 41.5 |

Table S9 The dioxygenase gene in the genome of *Sphingomonas* sp.SC-1

| Serial number | Gene ID | Sequence length（bp） | NR functional annotation |
| --- | --- | --- | --- |
| 01 | *gene*0122 | 1014 | 2-nitropropane dioxygenase |
| 02 | *gene*0867 | 1410 |  |
| 03 | *gene*0670 | 876 | Phytane - coA dioxygenase |
| 04 | pB_*gene*0062 | 915 |  |
| 05 | *gene*2920 | 1329 | Dioxygenase phthalate |
| 06 | *gene*2928 | 1362 | Polycyclic aromatic hydrocarbons cyclohydroxylated dioxygenase α subunit |
| 07 | *gene*2929 | 486 | Small subunit of angular dioxygenase |
| 08 | *gene*2970 | 480 | Protocatechuic acid 4, 5-dioxygenase β subunit |
| 09 | *gene*2971 | 357 |  |
| 10 | *gene*2972 | 351 | Protocatechuic acid 4, 5-dioxygenase α subunit |
| 11 | *gene*2975 | 399 |  |
| 12 | *gene*2976 | 843 | Protocatechuic 3, 4-dioxygenase |
| 13 | *gene*2984 | 1263 | The ferridoxin reductase component of carbazole 1, 9α-dioxygenase |
| 14 | *gene*3052 | 1044 | 4-hydroxyphenylpyruvate dioxygenase |
| 15 | *gene*1287 | 1212 | Nitric oxide dioxygenase |
| 16 | pC_*gene*0033 | 1230 | Phenoxybenzoate dioxygenase α subunit |
| 17 | pC_*gene*0035 | 960 | Phenoxybenzoate dioxygenase β subunit |
| 18 | pC_*gene*0073 | 891 | Catechol 1, 2-dioxygenase |


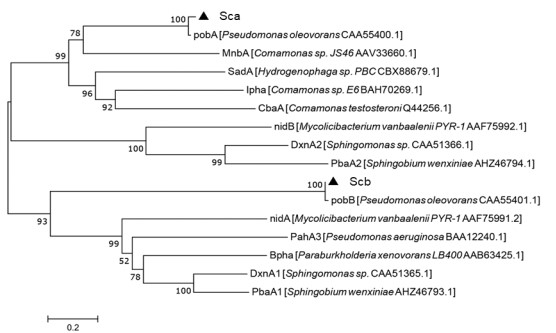


Fig. S11. Phylogenetic tree of Sca*,* Scb and other dioxygenases


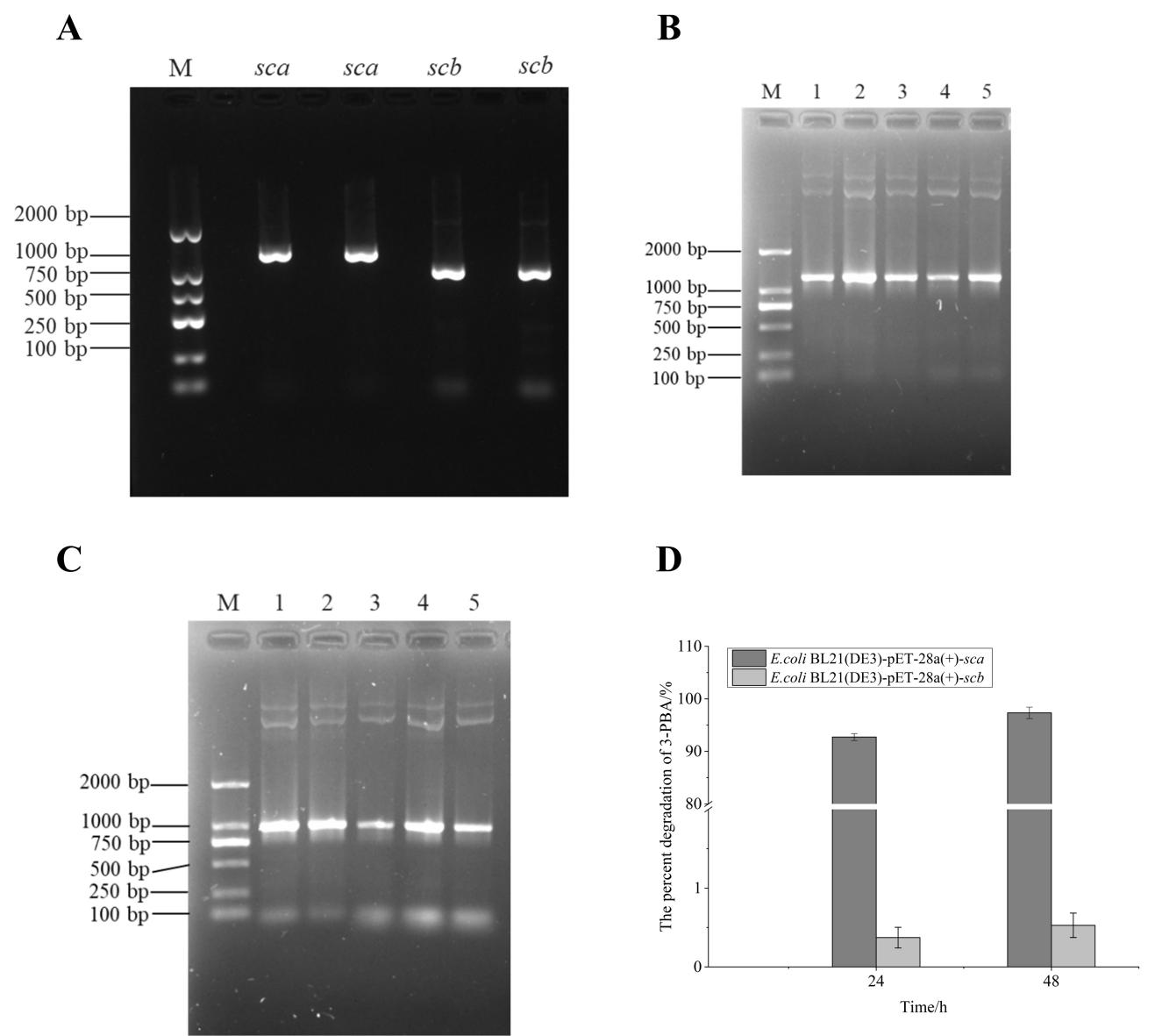


Fig. S12. Cloning and validation of *sca and scb*

A: Nucleic acid electrophoresis of *sca and scb*. B and C: Identification of the pET-28a(+)-*sca* (B) and pET-28a(+)-*scb* (C) recombinant plasmids. D: Percent degradation of engineered bacteria on 3-PBA.


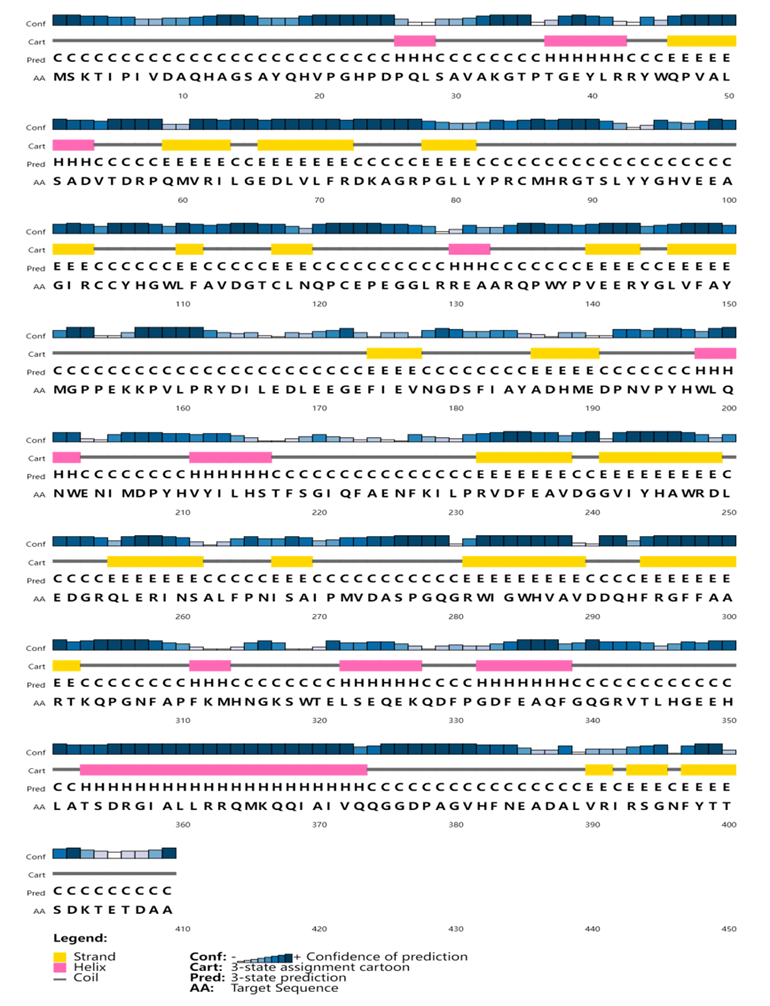

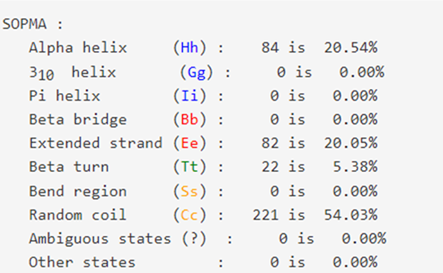


Fig. S13 The predicted secondary structure of Sca


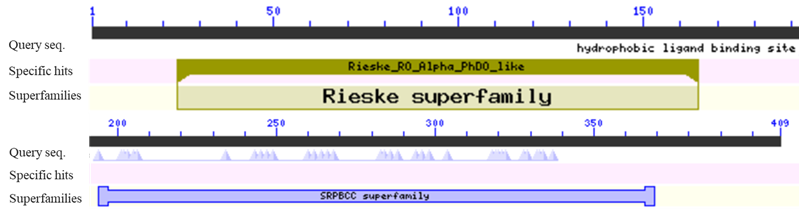


Fig. S14 The conserved domain analysis of Sca


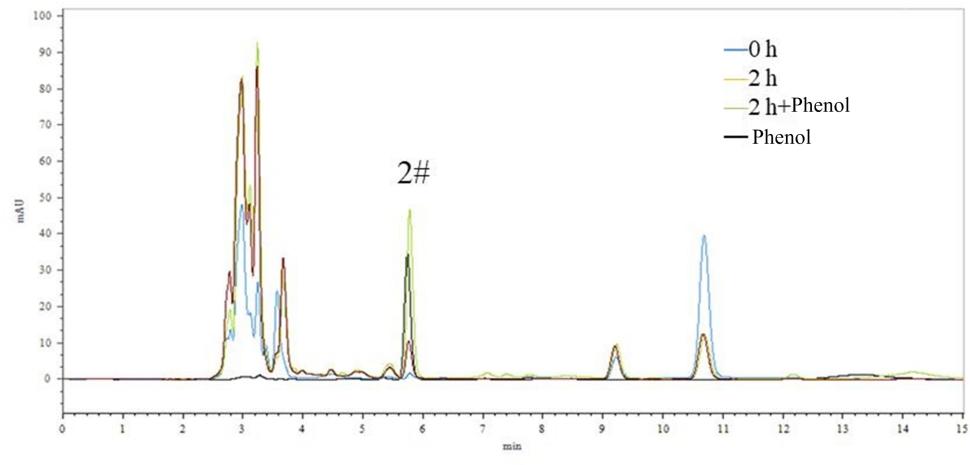


Fig. S15 HPLC analysis of the 3-PBA sample solution containing phenol added for the degradation of resting cells.

Table S10 Analysis of degradation rates for different substrates

| Substrate | Degradation rate/% |
| --- | --- |
| 4′-HO-3-PBA | 100 |
| 3′-HO-4-PBA | 100 |
| Phenol | ＜1 |
| Hydroquinone | ＜1 |
| Gallic acid | ＜1 |
| Protocatechuic acid | ＜1 |
| Flusulfaxide | ＜1 |
